# Land-use choices follow profitability at the expense of ecological functions in Indonesian smallholder landscapes

**DOI:** 10.1038/ncomms13137

**Published:** 2016-10-11

**Authors:** Yann Clough, Vijesh V. Krishna, Marife D. Corre, Kevin Darras, Lisa H. Denmead, Ana Meijide, Stefan Moser, Oliver Musshoff, Stefanie Steinebach, Edzo Veldkamp, Kara Allen, Andrew D. Barnes, Natalie Breidenbach, Ulrich Brose, Damayanti Buchori, Rolf Daniel, Reiner Finkeldey, Idham Harahap, Dietrich Hertel, A. Mareike Holtkamp, Elvira Hörandl, Bambang Irawan, I. Nengah Surati Jaya, Malte Jochum, Bernhard Klarner, Alexander Knohl, Martyna M. Kotowska, Valentyna Krashevska, Holger Kreft, Syahrul Kurniawan, Christoph Leuschner, Mark Maraun, Dian Nuraini Melati, Nicole Opfermann, César Pérez-Cruzado, Walesa Edho Prabowo, Katja Rembold, Akhmad Rizali, Ratna Rubiana, Dominik Schneider, Sri Sudarmiyati Tjitrosoedirdjo, Aiyen Tjoa, Teja Tscharntke, Stefan Scheu

**Affiliations:** 1 Centre for Environmental and Climate Research, Lund University, Sölvegatan 37, 22362 Lund, Sweden; 2 Department of Crop Sciences, Agroecology, Georg August University Göttingen, Grisebachstr. 6, 37077 Göttingen, Germany; 3 Department of Agricultural Economics and Rural Development, Georg August University Göttingen, Platz der Göttinger Sieben 5, 37073 Göttingen, Germany; 4 Soil Science of Tropical and Subtropical Ecosystems, Büsgen Institute, Georg August University Göttingen, Büsgenweg 2, 37077 Göttingen, Germany; 5 Bioclimatology, Georg August University Göttingen, Büsgenweg 2, 37077 Göttingen, Germany; 6 Institute of Social and Cultural Anthropology, Georg August University Göttingen, Theaterplatz 15, 37073 Göttingen, Germany; 7 Systemic Conservation Biology, Georg August University Göttingen, Berliner Str. 28, 37073 Göttingen, Germany; 8 German Centre for Integrative Biodiversity Research (iDiv) Halle-Jena-Leipzig, Deutscher Platz 5e, 04103 Leipzig, Germany; 9 Forest Genetics and Forest Tree Breeding, Büsgen Institute, Georg August University Göttingen, Büsgenweg 2, 37077 Göttingen, Germany; 10 Institute of Ecology, Friedrich Schiller University Jena, Dornburger-Str. 159, Jena 07743, Germany; 11 Department of Plant Protection, Faculty of Agriculture, Bogor Agricultural University Jalan Kamper Kampus IPB Darmaga, Bogor 16680, Indonesia; 12 Department of Genomic and Applied Microbiology and Göttingen Genomics Laboratory, Institute of Microbiology and Genetics, Georg August University Göttingen, Grisebachstr. 8, 37077 Göttingen, Germany; 13 Department of Plant Ecology and Ecosystems Research, Georg August University Göttingen, Untere Karspüle 2, 37073 Göttingen, Germany; 14 Department of Systematics, Biodiversity and Evolution of Plants, Georg August University Göttingen, Untere Karspüle 2, 37073 Göttingen, Germany; 15 Forestry Faculty, University of Jambi, Campus Pinang Masak Mendalo, Jambi 36361, Indonesia; 16 Forest Resources Inventory and Remote Sensing, Bogor Agricultural University, Kampus IPB Darmaga, Bogor 16680, Indonesia; 17 JF Blumenbach Institute of Zoology and Anthropology, Animal Ecology, Georg August University Göttingen, Berliner Str. 28, 37073 Göttingen, Germany; 18 Biodiversity, Macroecology & Conservation Biogeography, Georg August University Göttingen, Büsgenweg 1, 37077 Göttingen, Germany; 19 Department of Soil Science, Faculty of Agriculture, Brawijaya University. Jl. Veteran 56 Malang, East Java, 65145, Indonesia; 20 Forest Inventory and Remote Sensing, Burckhardt Institute, Georg August University Göttingen, Büsgenweg 5, 37077 Göttingen, Germany; 21 Conservation Biology Division, Institute of Ecology and Evolution, University of Bern, Baltzerstrasse 6, CH-3012 Bern, Switzerland; 22 Department of Plant Pests and Diseases, Faculty of Agriculture, University of Brawijaya. Jl. Veteran Malang, East Java 65145, Indonesia; 23 SEAMEO BIOTROP Regional Center for Tropical Biology, Jl. Raya Tajur km 6, Bogor 16134, Indonesia; 24 Faculty of Agriculture, Tadulako University, Jl. Soekarno Hatta km 09 Tondo, Palu 94118, Indonesia

## Abstract

Smallholder-dominated agricultural mosaic landscapes are highlighted as model production systems that deliver both economic and ecological goods in tropical agricultural landscapes, but trade-offs underlying current land-use dynamics are poorly known. Here, using the most comprehensive quantification of land-use change and associated bundles of ecosystem functions, services and economic benefits to date, we show that Indonesian smallholders predominantly choose farm portfolios with high economic productivity but low ecological value. The more profitable oil palm and rubber monocultures replace forests and agroforests critical for maintaining above- and below-ground ecological functions and the diversity of most taxa. Between the monocultures, the higher economic performance of oil palm over rubber comes with the reliance on fertilizer inputs and with increased nutrient leaching losses. Strategies to achieve an ecological-economic balance and a sustainable management of tropical smallholder landscapes must be prioritized to avoid further environmental degradation.

## Abstract (web summary)

Small-scale farmers in Southeast Asia are increasingly turning to monocultures of oil palm and rubber to maximize income. Clough and colleagues demonstrate that this land-use change in Indonesia comes at a cost to a wide array of ecosystem functions and biodiversity.

## Graphical abstract

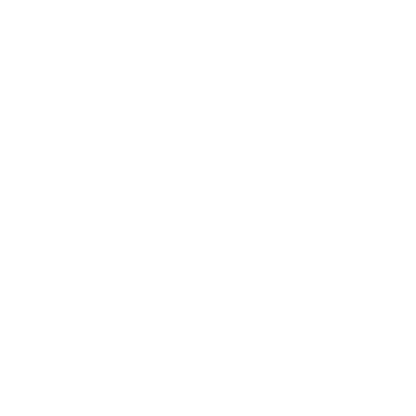

Large expanses of lowland tropical rainforest have been converted to large-scale commercial plantations or small-scale mosaic agricultural landscapes^1^, in which fragments of forests are surrounded by a mixture of settlements, monocultures and mixed-species land uses. While smallholder-dominated mosaic landscapes often retain natural resources and combine land uses that support complementary ecosystem functions, services and benefits^2^,^3^, these are subject to trade-offs and synergies. For instance, an immediate effect of the production of food and other raw materials on economic benefits^4^ could drive increases in crop production and associated returns from the land at the expense of other ecological functions. Further, land-use intensification, conversion of semi-natural habitat remnants and specialization on a few cash crops remain pervasive^5^. Studies combining empirical evidence on land-use dynamics, economic benefits, biodiversity and ecological functions in smallholder systems are scarce but essential to better understand these dynamics.

The present study aims at quantifying land-use dynamics and their drivers, as well as economic and ecological impacts of land-use choices in smallholder-dominated tropical landscapes in Sumatra, Indonesia (Fig. 1a), using a unique multidisciplinary data set collected in a collaborative project by over 20 research groups. Originally covered by sparsely populated rainforest, large parts of the lowlands now consist of large-scale oil palm, *Acacia* plantations, and small-scale smallholder-dominated mosaics of forest remnants, jungle rubber (rubber-enriched secondary forest^6^ and rubber monocultures) and oil palm monocultures (Fig. 2a–d; see Supplementary Note 1 for historical and institutional background). While expansion of large-scale industrial plantations of oil palm in the region have raised much environmental concern, dynamics in smallholder land-use and their consequences are less well known. This is despite smallholder-managed land making up the largest share of agricultural land, even among so-called ‘estate crops' such as rubber and oil palm^7^. We expect the most productive and profitable agricultural land-use types to be increasing, and that underlying ecological-economic trade-offs lead to reductions not only in biodiversity, but also in key ecological functions underpinning ecosystem services such as climate regulation and water quality. We assessed land-use, profitability, agricultural inputs and outputs for 464 smallholder households from 45 villages in Jambi province, Sumatra (Supplementary Fig. 1), and attitude to risk was quantified for a subsample of farmers. Land-use transitions over the past 20 years were assessed at household level with survey data, and at regional level using land-use classification inferred from remote sensing. For each of the studied land-use types (rainforest, jungle rubber, rubber and oil palm plantations), we empirically assessed biodiversity, ecological functions and ecosystem services in 32 core study sites (eight per land-use type, Supplementary Fig. 1). First, we evaluated biodiversity with (i) three indicators for naturalness index: forest bird species, indigenous tree species and absence of the ten common invasive weed species, (ii) local species richness across important plant, vertebrate, invertebrate, protist and prokaryote groups and (iii) plant genetic diversity. The former is a proxy for the potential to contribute to nature conservation, while the others have been shown to support ecosystem functioning^8^. Second, we assessed stability in micro-climatic conditions on the basis that a buffering from extreme conditions is beneficial for biodiversity and ecosystem functioning^9^. Third, we quantified leaf litter decomposition, soil microbial functioning and nutrient leaching in the soil as indicators of regulating services through sustainability of soil fertility and ground-water quality. Fourth, we measured harvested yield, net primary production (NPP) and ecosystem carbon stocks as indicators of both provisioning (yield) and regulating (carbon sequestration) services. We find that the more profitable oil palm and rubber monocultures replace forested systems that play a key role in supporting biodiversity end ecological functions. Oil palm is profitable and attractive, but degrades soil quality and causes nutrient leaching. Finding strategies to balance ecological and economic functions in these landscapes, including a more sustainable management of smallholder oil palm, is required to avert further environmental problems.

## Results

### Productivity, inputs and profitability

Farms were characterized by a low diversity (high specialization) of cultivated crops (Supplementary Fig. 2), especially in the transmigrant villages. [Table t1]Rubber and oil palm were most prevalent, being cultivated by 82 and 35% of smallholder farmers, respectively (Supplementary Table 1). Rubber was predominantly grown in monoculture, as only 17% of visited rubber plots could be categorized as jungle rubber. Cultivation of other crops was much less common and undertaken only in small plots (Supplementary Table 1). Distribution of land ownership was highly uneven, with 50% of the land area being held by about 10% of the farm households (Supplementary Fig. 3). The household survey showed that oil palm was managed much more intensively with herbicides, and soil amendments (that is, chemical fertilizers and lime) than rubber, but required less labour (see Fig. 3a,b,d; statistical results and summary statistics in Table 2 and Supplementary Table 2). Hence, oil palm cultivation resulted in a high gross margin per labour unit (high labour productivity), but lower gross margin per land unit (low land productivity), compared with rubber plantations (Fig. 3c,d). On the other hand, compared with oil palm, both jungle rubber and monoculture rubber were labour-intensive, with a low labour productivity (Fig. 3b,d), due to rubber being harvested around five times a week, compared with once in every two weeks for oil palm. This difference was crucial to explain the land-use changes in Jambi, as it helps the labour-constrained smallholders expand their farm by incorporating oil palm in the crop-portfolio. Rubber plantations had a higher land productivity than jungle rubber (Fig. 3c). In the core plots, maximum and mean rubber yield in monoculture plantation was four and two times the yield in jungle rubber, respectively (Fig. 4g). Oil palm plantations were cultivated by more risk-averse farmers than jungle rubber (Holt–Laury values; LR-test; *P*=0.044; Fig. 3e), whereas farmers with monoculture rubber showed intermediate levels of risk-taking. Transmigrant villages differed from non-transmigrant villages in having higher fertilizer costs, in particular in oil palm, and in having a larger share of family labour over hired labour in jungle rubber and rubber plantations, and higher gross margins (Table 2 and Supplementary Table 2).

### Regional and household-scale land-use changes

**Figure 1:**
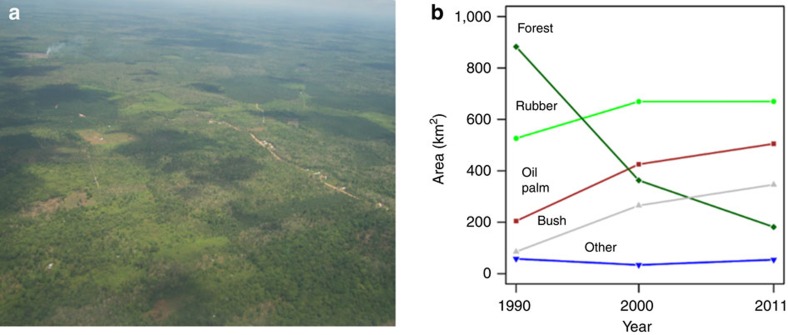
Land-use change in smallholder-dominated mosaic agricultural landscapes. Aerial photo (Photo: Heiko Faust) (**a**) and changes in the land-use composition (**b**) of the studied landscapes around the national park Bukit Duabelas and the Harapan Forest Restoration concession in Jambi Province, Indonesia from 1990 to 2011, based on land-use classification inferred from remote sensing. Rainforest (dark green diamonds), rubber (light green filled circles), oil palm (red squares), shrub/bushland (grey up-pointing triangles), and ‘others' (blue down-pointing triangles), which includes amongst others food crops, timber and fruit tree plantations. See Table 1 for the 1990–2011 land-use change matrix.

Between 1990 and 2011, unprotected forest within the study area decreased by more than 75% (Fig. 1b, Supplementary Fig. 4). For comparison, in the same period forest decrease was only 13% inside the two protected areas, Bukit Duabelas National Park and Harapan Rainforest restoration concession, where our forest core plots were located. In the same period, rubber increased by 30%, oil palm by 150%, and shrub/bushland, which were mostly fallow lands awaiting planting with rubber or oil palm, by 300% (Fig. 1b). Over 80% of farm plots belonging to the surveyed households were acquired or established after 1990 (Supplementary Fig. 5), confirming significant expansion of area under smallholder-managed plantation crops in the last two decades. Farmers reported that oil palm and rubber were developed from shrub/bushland (33% of oil palm, 27% of rubber) and direct deforestation (14% of oil palm, 32% of rubber), the latter being more commonly reported in the autochthonous villages (Supplementary Fig. 6). The remote-sensing data suggested that shrub/bushland was an intermediate state, with much of the rainforest losses being due to conversion to both oil palm and rubber (Table 1). There is still apparent potential for considerable cultivation expansion as one-fifth of the farmers possessed uncultivated fallows in 2011–2012 (mostly shrub/bushland, Supplementary Table 1).

### Biodiversity

Naturalness was highest in forest, and successively decreased in jungle rubber, rubber plantation and oil palm plantation (Fig. 4a, summary statistics and statistical results in Table 2 and Supplementary Table 3). Overall, plot-scale species richness was higher in forest and jungle rubber than in the monocultures (Fig. 4b), but individual taxa responded differently (Table 2). Plant, bird, termite, litter invertebrate and protist richness decreased from forest and jungle rubber to monocultures, while ant and archaeal richness did not differ among land-use systems, and bacterial richness was higher in the monocultures. Genetic diversity, assessed for ten dominant plant species in each plot, was higher in forests and jungle rubber than in the two monocultures (Fig. 4c).

### Ecological functions

Stability in micro-climatic conditions (temperature and humidity in air and soil) was highest in the forest, lower in the jungle rubber and lowest in the rubber plantations, with values for oil palm intermediate between rubber and jungle rubber (Fig. 4d, summary statistics and statistical results in Table 2 and Supplementary Table 3). Soil microbial biomass, microbial decomposer activity and leaf litter were similar in forest and jungle rubber but significantly lower in monocultures (Fig. 4e). Nutrient-leaching fluxes were higher in the fertilized oil palm than in the other three land-use types (Fig. 4f). Yield, measured as harvested biomass, was highest in oil palm, intermediate in rubber and lowest in jungle rubber (Fig. 4g); we assumed no extraction from forests. Fertilization-driven soil biochemical indicators were also higher in oil palm than in the other land-use types (Supplementary Fig. 7). Values of both net primary productivity (excluding yield) and carbon stocks were highest in forest, intermediate in jungle rubber and lowest in monocultures (Fig. 4h,i). Carbon (C) stocks were equally distributed amongst the plant biomass and soil organic C in forest and jungle rubber. In the monoculture plantations, C stocks in plant biomass were much lower than in forested systems, with much less marked differences for soil organic C stocks, which were very variable even within the same land-use type (Fig. 4i).

### Economic-ecological trade-offs

**Figure 2:**
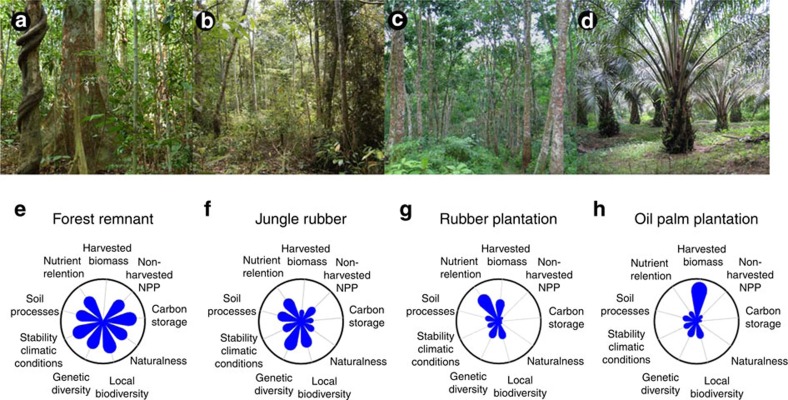
Land-uses and associated ecological functions. Forest remnants (**a**,**e**), jungle rubber (**b**,**f**), rubber plantation (**c**,**g**) and oil palm plantation (**d**,**h**). Ecological functions are represented as flower diagrams. For each function, the minimum (circle centre) is the 5th quantile and the maximum (circle edge) is the 95th quantile of the standardized ecosystem function indicators, observed in a plot of any land-use types. The outer edge of the flower petals indicates the estimate for the aggregate ecosystem function in a given land use relative to these minima and maxima. Because the importance of functions may differ between stakeholders, unweighted values are presented. Photo credits: Katja Rembold (**a**), and Yann Clough (**b**–**d**).

The trade-offs between ecological functions incurred by choosing one land-use over another are illustrated in a standardized manner in Fig. 2, and are also reflected in the ecological function correlation matrices (Supplementary Fig. 8). When considering ecological functions across a forest—agroforest—monoculture plantation sequence, production of harvested biomass increased, but most other functions decreased. Nutrient retention, calculated as the additive inverse of nutrient leaching, was the only function for which rubber monocultures, usually unfertilized in smallholder landscapes, attained similarly high relative values as forest and jungle rubber.

## Discussion

The persistence of biodiversity and ecosystem-service delivery in human-dominated tropical mosaic landscapes depends on land-use dynamics and the contribution of the dominant land uses to bundles of ecosystem services and local benefits, where significant trade-offs may be expected between economic and ecological functions. In smallholder-dominated landscapes of lowland Sumatra, forest cover has diminished drastically over the past 20 years and current land-use choices favour the adoption of the most profitable monocultures. These changes led to higher crop production and incomes among smallholders, but were accompanied with declines in multiple ecological functions directly related to biodiversity conservation, climate regulation and water quality.

The province of Jambi is a model of crucial dependency on its agricultural sector. In 2013, approximately half of the workforce was employed in the agricultural sector, a share which has not changed much over the past four years, while the total population in the province is increasing^7^,^10^. Rural poverty is low in Jambi (7%) in comparison with national urban-poverty value (14%). This is true in both absolute terms and in relation to urban poverty^10^, making the Jambi agricultural sector attractive for migrants. Between 1990 and 2010, the population of Jambi increased from 2 to 3 million, and that of the five regencies constituting our study area from 0.8 to 1.4 million^10^. Increased numbers of smallholders as well as increased area of large-scale plantations has gradually reduced the area of accessible farmland for smallholders. The increase in land scarcity has several effects: extensification to secure land, forced agricultural intensification as farmers' subsistence strategies shift from extensive ‘slash-and-burn' cultivation to cash-crop production, and increased agricultural transition^11^.

In agreement with previous studies^11^,^12^, we found that the total area under cultivation had increased, mainly due to the conversion of forest to oil palm and rubber. Deforestation, especially of near-primary forest, causes biodiversity losses that are impossible to compensate with other land uses, which is clearly visible from all three measures of biodiversity used in the present study. Besides the local loss in naturalness and biodiversity, the regional persistence of species even in larger, protected forest fragments (such as the Harapan Rainforest where there was a more stable forest cover, Supplementary Fig. 4) may be jeopardized in the long-term by increasing isolation from other forested habitats and by reducing connectivity of the landscape matrix following monoculture establishment^2^. With low values of naturalness and biodiversity of conservation-relevant groups, rubber and oil palm monocultures cannot contribute to the maintenance of the characteristic fauna and flora of the studied landscapes in general^13^, unless effective regional planning achieves the combination of high yields under monocultures with land set aside for forest regrowth^14^,^15^. Jungle rubber is associated with intermediate levels of biodiversity, but its usefulness for conservation is impeded by low yields and poor economic performance, which may potentially lead to increased deforestation elsewhere.

**Figure 3:**
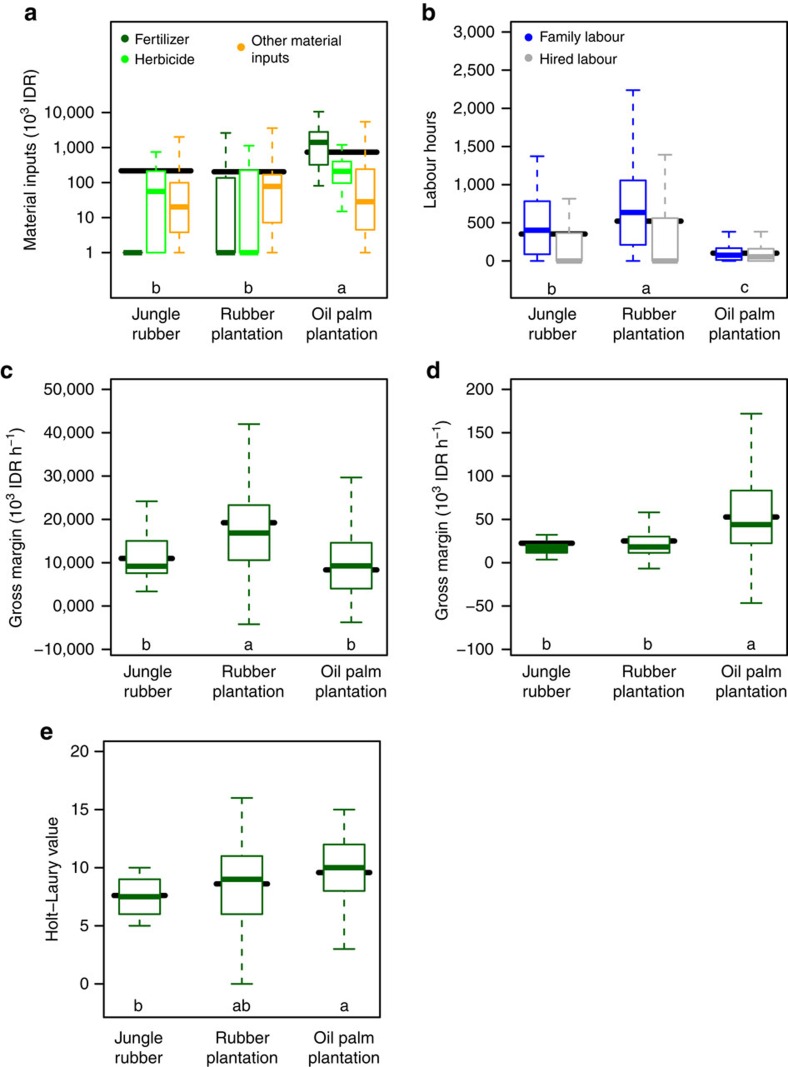
Economic functions for three agricultural land-uses. Measures for agricultural input use (**a**–**b**), profitability functions (**c**–**d**), and farmer risk aversion (**e**) in three agricultural land-uses (Jambi, Indonesia): material inputs (**a**), labour inputs (**b**), gross margin per hectare (**c**) in units of 10^3^ Indonesian Rupiah (IDR) ha^−1^ yr^−1^ and gross margin per labour unit (**d**) in units of 10^3^ IDR h^−1^. Farmers risk aversion (**e**) is measured with a Holt–Laury lottery, higher values indicate higher risk aversion. Summary statistics of all variables are in Supplementary Table 2. Boxplots indicate the lower quartile, median and upper quartile, with whiskers extending to the most extreme data point that is no more than 1.5 times the interquartile range from the edge of the box. Horizontal bars indicate the estimated mean indicator variable value; letters indicate significant differences (F/Wald-tests; *P*<0.05) in these linear model (**c**–**e**) and linear mixed model (**a**–**b**) estimates between the land-use types. Boxplots within land-use (from left to right) are labelled within the panels (from top to bottom and left to right).

Jungle rubber was formerly the main rubber production system^6^, but low land and labour productivity (Fig. 3c,d) explain why jungle rubber decreased in area, while both monocultures increased (Table 1). Rubber and oil palm plantations were complementary^16^, in that rubber plantations had high labour productivity and oil palm high return-to-labour. Although the per hectare land productivity was comparable between monoculture rubber and oil palm plantations, households facing labour constraints could increase and diversify their farm income by adopting oil palm, which required relatively less involvement of labour. In interviews of smallholders, respondents stated that they viewed oil palm as an easier crop to cultivate. The risk-averseness of farmers cultivating oil palm over farmers owning jungle rubber may seem surprising given the flexibility of the agroforestry systems^17^, but suggests other causes of oil palm expansion besides the attractiveness of higher and quicker returns. Substantial economic benefits of the expansion of monoculture cultivation were apparent from our data and are visibly linked to increased human welfare in the region, as is the case elsewhere^14^,^18^. Focusing mainly on contribution to average farmer income may mask that human welfare is not limited to economic variables, and that the impact of land-use change may affect different persons differently, depending on gender, ethnicity, social and economic status^19^. For instance, our results support differences in agricultural inputs and profitability between systems for transmigrant and non-transmigrant villages. The data analysed in this study do not allow for a full assessment of impacts of land-use changes on human wellbeing. However, welfare impacts are found strongly linked to the farmer heterogeneity and differential factor (especially human labour) endowment of the farm-household^20^, indicating potentially negative implication of plantation expansion on economic equality. The degree to which these developments benefit the whole population thus is uncertain and the inequality in holding size—with 10% of the farmers holding over 50% of the land area—suggests significant disparities^21^. Besides these potential socio-economic caveats, our study highlights pervasive negative side effects on ecological functions and the natural capital and ecosystem services they support.

**Figure 4:**
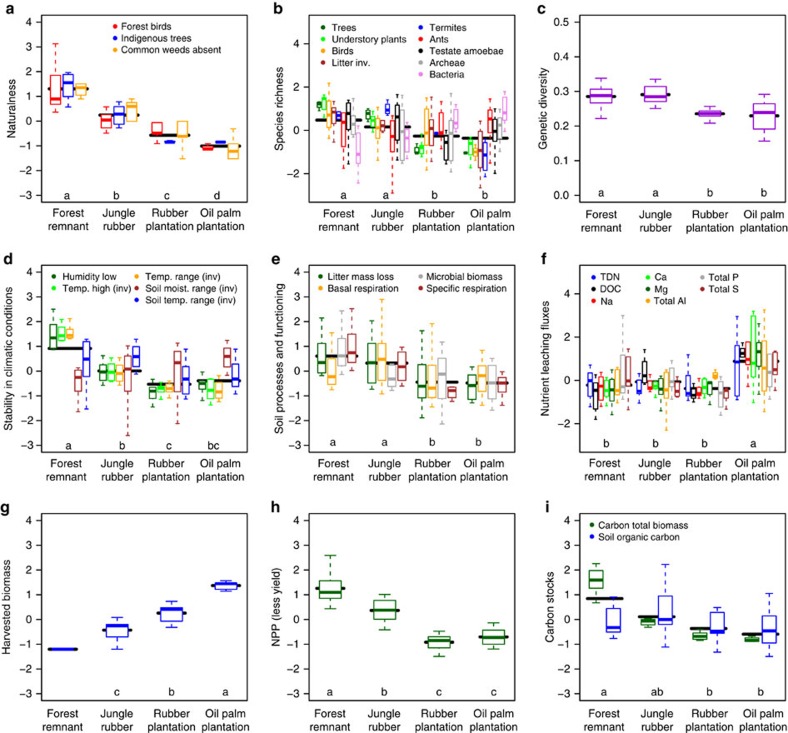
Multiple aggregate ecosystem functions and their indicators. Naturalness (**a**), observed local species richness (**b**), genetic diversity of plants (**c**), stability in climatic conditions (**d**), soil processes and functioning (**e**), nutrient leaching fluxes (**f**), yield/harvested biomass (**g**), NPP minus exported harvest (**h**) carbon stocks (**i**). Indicators for naturalness (**a**) are: proportion forest species among bird communities, proportion indigenous tree species, proportion common weed species present. Indicators for biodiversity/species richness (**b**) are: number of species/OTU of trees, understory plants, birds, litter invertebrates, termites, ants, testate amoebae, archaea and bacteria recorded per plot. Indicator for genetic diversity of plants (**c**) is Shannon diversity based on 10 individuals for 10 dominant plant species per plot. Indicators for stability in climatic conditions (**d**) are: lower 5% percentile for air humidity, higher 95% percentile for air temperature (additive inverse), and ranges (additive inverse) between percentiles 5 and 95% for air temperature, soil moisture range and soil temperature. Indicators for soil processes and functioning (**e**) are: litter mass loss after 6 months, soil microbial biomass, soil basal respiration and soil specific respiration. Indicators for nutrient leaching fluxes (**f**) are: total dissolved nitrogen (TDN), dissolved organic carbon (DOC), Na, Ca, Mg, total Al, total P and total S. Indicators for carbon stocks (**i**) are: estimated carbon in total plant biomass and SOC. All variables were standardized to allow joint plotting; summary statistics of raw variables are in Supplementary Table 3. Boxplots indicate the lower quartile, median and upper quartile, with whiskers extending to the most extreme data point that is no more than 1.5 times the interquartile range from the edge of the box. Horizontal bars indicate the estimated mean indicator variable value; letters indicate significant differences (F/Wald-tests; *P*<0.05) in mean levels of these linear model (**c**,**g**,**h**) and linear mixed model (**a**,**b**,**d**–**f**,**i**) estimates between the land-use types. Boxplots within land-use (from left to right) are labelled within the panels (from top to bottom and left to right).

While oil palm plantations have attracted more attention than rubber for their negative environmental impacts, we show that both monocultures perform similarly in terms of most ecological functions and services, despite the crop plants and the resulting vegetation structure being very different. The lower taxonomic and genetic plant diversity (Fig. 4a–c), simpler vegetation structure and more variable microclimate of monocultures (Fig. 2a–d) in comparison with forested systems were concordantly associated with low species richness of birds, invertebrates and protists, as expected. However, similar plot-scale diversity of ant and archaea diversity across systems, and higher bacterial diversity in monocultures than forested systems showed that communities were not always simpler in monocultures. The detailed linkages between biodiversity and ecosystem functions assessed in our study are still being investigated, yet first results show that the observed changes in biodiversity are accompanied by strongly altered soil food webs^22^, leading to equally strong alteration of ecosystem functioning and soil processes (Fig. 4e). As examples, decomposition and specific respiration, a key process controlling carbon and nutrient cycling, shifted from being large in forest and jungle rubber with high biodiversity and stable abiotic factors to being reduced in oil palm and rubber monocultures. In contrast to findings of previous study, where a dominant termite species maintained similarly high decomposition rates in oil palm as in forest^23^, our results show that decomposition of tree leaf litter in monoculture systems was slower (*ca*. 61% less mass loss, Table 2 and Supplementary Table 3) compared with forest and jungle rubber. These shifts are paralleled with other changes in soil biochemical characteristics. In a recent pan-tropic study, including Jambi Province, lowland forest conversion to smallholder oil palm and rubber plantations was associated by the loss of up to 50% of stored soil organic carbon (SOC) in the original forest soils^24^. These SOC losses were contributed by an increase in soil erosion^25^, a decrease in NPP and thus in organic matter input (Fig. 4e) and changes in abiotic conditions (Fig. 4d) that altered leaf litter decomposition (Fig. 4e) in rubber and oil palm monoculture.

The fertilized oil palm plantations stand out as having very high nutrient-leaching fluxes. Amongst the environmental aspects which autochthonous residents most frequently associated with oil palm expansion were periodic decreases in water quality and quantity^26^, resulting in scarcity of water for drinking, bathing and washing clothes (see Carlson *et al*.^27^ for freshwater data from Kalimantan). In oil palm, fertilization is an important management practice, without which decline in soil fertility with years of cultivation after deforestation would be inevitable^28^. Nitrogen fertilization in oil palm plantations was associated with high nutrient leaching (Fig. 4f) which may have negative impacts on ground-water quality. In addition, N-oxide emission from the soil may have increased as can be inferred from the increased soil ^15^N natural abundance signatures in oil palm plantations (Table 2), which is a result of isotopic fractionation from soil processes producing gaseous N (nitrification and denitrification) leaving isotopically-enriched soil N behind^29^. Given the low acid-buffering capacity of Acrisol soils (Supplementary Table 3), which cover 50% of the land area in Sumatra, continued N fertilization will lead to more deleterious effects (for example, further increases in aluminium solubility and base cation leaching losses and decrease in soil phosphorus availability, Fig. 4f and Supplementary Table 3) rather than just increase N availability, unless lime is applied^30^. Oil palm plantations will increasingly be dependent on fertilization and liming, which incur additional costs to smallholders unless sustainable management practices are employed. Thus, it is essential that management trials be tested on-site to screen for practices that will yield optimum benefits (for example, harvest and profit) with less nutrient losses, that is, by combining better fertilization management and improving nutrient retention efficiency in the soil.

The performance of agroforestry systems such as jungle rubber for multiple ecological functions, aside from providing income, suggests that they could in principle serve to sustain both ecological and economic functions. However, in our studied landscapes, smallholder jungle rubber produced less and generated less income than monocultural rubber (Fig. 3c,d). A combination of monocultures and reforestation may therefore, at least theoretically, be more efficient in combining agriculture and conservation. Unless land-use policy options provide economic incentives for their preservation, primary forests, secondary forest and jungle rubber have little future in smallholder-dominated landscapes despite their contribution to biodiversity and ecosystem services. In principle, however, opportunities for combining agriculture and conservation already exist. A priority region called the RIMBA (RIau, JaMBi and Sumatra BArat) Integrated Ecosystem to the north of our study area, which straddles Jambi as well as Riau and West Sumatra, has been designated by the Indonesian Ministry of Public Works as a demonstration area for implementing ecosystem-based spatial planning^31^, which could facilitate the allocation of land to forested land-uses. Proper implementation of the REDD+ (reducing emissions from deforestation and forest degradation and the role of conservation, sustainable management of forests and enhancement of forest carbon stocks in developing countries) program may open up economic incentives for communities to reforest. The positive correlations of C stocks and non-harvested NPP with other ecological functions (Supplementary Fig. 8) suggest that this would benefit multiple ecosystem services. However, at present, concrete incentives to conserve natural capital and ecosystem services in smallholder-dominated landscapes are absent^32^, and REDD+ may not be economically attractive on mineral soils as an alternative to oil palm development^33^. A cornerstone of the economic development plan for Sumatra^34^ is the intensification of smallholder rubber and oil palm, which could be seen on the one hand as an opportunity to achieve higher production levels on less land. On the other hand, it could simply reinforce negative environmental impacts of monocultures, without gains in productivity being translated to increases in land spared for forests. The question is whether increasing wealth, locally, regionally and nationally, could in the long-term place improvement of environmental performance higher up on the agenda, which could then lead to the development and enforcement of agri-environmental regulations and incentives^35^. Recent findings suggest that this will depend on the strengthening of environmental governance^36^, and, especially if agriculture is not to be segregated from ecosystem-service generation, on rewarding land managers for increased ecosystem services delivery^37^.

## Methods

### Study region, households and study sites

The province of Jambi, on the island of Sumatra, covers a total land area of 5 Mha (million hectares). We focused on the five regencies that comprise most of the lowland, non-peat smallholder systems: Sarolangun, Bungo, Tebo, Batanghari and Muaro Jambi^10^. From a questionnaire-based farm household survey, covering 701 smallholder farmers randomly selected from 45 villages (covering both autochthonous/transmigration villages), we used the data of 464 smallholder respondents whose main parcel of cropland we have visited and categorized as either jungle rubber (*n*=33), monoculture rubber plantation (*n*=162) or monoculture oil palm plantation (*n*=269). These respondents were independent smallholders, with the exception of 50 oil palm farmers which were associated with an oil palm company during establishment. The interviews were conducted in the second half of 2012. For the ecological studies, we selected within the study region two landscapes, ‘Harapan' and ‘Bukit Duabelas', with loam and clay Acrisol soils, respectively (see details below). In each landscape, we selected four 50 × 50 m replicate plots for each land-use systems: primary degraded forest, jungle rubber, monoculture rubber plantation (10–17 years old) and monoculture oil palm plantation (12–16 years old). Most measurements were conducted in five 5 × 5 m subplots within each plot. Research permits are listed in Supplementary Table 4. Forest plots were situated in the Bukit Duabelas National Park and the Harapan Rainforest Restoration concession (PT REKI).

### Household survey

The aim was to assess the micro-level determinants of recent changes in land-use in the lowlands of Jambi Province, as well as their impacts on smallholder welfare. We examined the adoption patterns, compared the economic profitability of the different land-uses. A stratified random sampling approach was followed, fixing the number of districts per regency and the number of villages per district *a priori*. A total of forty villages—two rural villages per district, four districts per regency in each of five regencies—were selected randomly. In addition, five villages were selected near to the Bukit Duabelas National Park and the Harapan Rainforest Restoration concession, where ecological studies were carried out. A complete list of households that are involved in farming activities during the last five years was prepared from each of the selected villages. Population size ranged from about 100 to >2,000 households per village. To reduce under-representation of households residing in larger and over-representation of households residing in smaller villages, we divided the randomly selected villages into four quarters based on population size. Six households were selected from each of the 10 villages in the lowest size quartile, 12 households per village from the second quartile, 18 households per village from the third and 24 households per village from the largest village size quartile, resulting in a total sample of 600 households. From each of the five additionally selected villages, about 20 households were randomly selected for the survey. Details of sampling with a list of sampled villages and number of sampled households per village are described by Faust *et al*.^38^. Information on crops and livestock managed by the households in 2012, socio-demographic characteristics, details of off-farm income, asset status and expenditures on food and non-food items were obtained in the survey. Due to significant socio-economic heterogeneity existing in the study area, farmer access to factors of production was variable, resulting in adoption of unique cropping patterns. The median of operating landholding size was 2.5 hectares (ha). The Basic Regulations on Agrarian Principles and Government Regulation of Indonesia stipulate ceiling for landholding size that is region-specific. In Jambi, an agricultural household can possess up to 20 ha of land for cultivation. About 99% of sample farmers possess land below this ceiling level. The key findings do not vary significantly even if we exclude the upper 1% from the analysis.

The questionnaire sections on current land uses and changes over the years and input–output data from all major crop plots are relevant for the present analysis. Inputs, outputs and income details were collected for the 1-year period preceding the survey of 2012. The questionnaire was pre-tested several times to ensure consistency and accuracy of the data, and was translated to Bahasa Indonesia using the service of a professional translating agency in Jambi. Informed consent was obtained from all subjects, and the University of Göttingen did not require ethics board approval for these socio-economic surveys.

Fluctuations in market prices for palm oil and rubber could limit the assessment of the relative profitability of these crops, but the parallel patterns of their market prices (Supplementary Fig. 9) suggest that interpreting past land-use decisions on the basis of current profitability is unlikely to be biased by their temporal changes in prices.

### Farmer attitudes to risk

Farmers' risk aversion was measured with a Holt–Laury lottery^39^. A higher Holt–Laury value indicates a more risk-averse farmer. The majority of the farmers from the household survey were invited to the experiment for measuring their risk attitude, with voluntary participation resulting in 10, 80 and 29 observations for jungle rubber, rubber and oil palm plantations, respectively, covering 33 of the previously selected 40 villages.

### Land-use classification and land-cover change

Landsat satellite images of the study area from TM 1989–1990, TM/ETM+ 1999–2001 and TM/ETM+2009–2011 were used to produce less cloud cover mosaics of images for the years 1990, 2000 and 2011, respectively. Due to the high cloud coverage in the study area, satellite images acquired in a period of±2 years was considered for each acquisition. The less cloud mosaic for each acquisition was produced after histogram matching for the bands 5, 4 and 3. Land-use/land-cover (LULC) maps were produced for each acquisition by visual interpretation as defined in GOFC-GOLD^40^. On-screen digitation was conducted with a band composite of 5, 4, 3 for the red, green and blue combination, respectively. The classification was enhanced by assisting the visual interpretation with higher resolution RapidEye imagery from 2013 for the 2011 acquisition, and with the use of the guidelines for land-cover mapping^41^ published by the Indonesian Ministry of Forestry (MoF) and with the expert knowledge for all the acquisitions. The initial maps was produced based on 23 classes considered by the MoF, and the additional target land-use systems such as jungle rubber, rubber and oil palm plantations. For the purpose of this study, LULC classes were aggregated into: forest, rubber, oil palm, shrub/bush and other. Jungle rubber and rubber plantation were combined because they could not be well distinguished, and this combination resulted in significant improvements in the accuracy of the overall classification. On the basis a ground-truth validation with 298 samples of systematically selected points, the overall accuracy of the classification was 78.2%. A change matrix was derived for the acquisitions of 1990 and 2011 by overlaying the LULC maps. The changes between the two acquisitions were expressed in percentage of the total area.

### Experimental design of core plots for the ecological studies

The study region (Supplementary Fig. 1) was delineated into two main landscapes that are both on heavily weathered soils but mainly differed in texture: loam and clay Acrisol soils^42^. In each landscape, four land-use systems were studied: rainforest, jungle rubber, and smallholder monoculture plantations of rubber (7–17 years old) and oil palm (9–16 years old). The loam Acrisol landscape (between 1.79° S, 103.24° E and 2.19° S, 103.36° E) is located ∼60 km south of Jambi city, and the clay Acrisol landscape (between 1.94° S, 102.58° E and 02.14° S, 102.85° E) is located ∼110 km west of Jambi city. Acrisols are characterized by clay translocation in the soil profile, low-effective cation exchange capacity (ECEC; <24 cmol charge kg^−1^ of clay within 0.5 m depth) and low base saturation (<50% within 0.5–1.0 m depth). The mean annual temperature was 26.7±0.2 °C and mean annual precipitation was 2,235±385 mm (1991–2011; data from the Indonesian Meteorological, Climatological and Geophysical Agency at a meteorological station located at the Sultan-Thaha Airport in Jambi). For each land use in each landscape, four replicate plots of 50 × 50 m were selected. Minimum distance between plots was 116 m, and altitude varied between 35 and 95 m above sea level. Forests represent selectively logged-over old-growth forest, which is equivalent to ‘primary degraded forest' as classified by Margono *et al*.^43^ Jungle rubber represents a smallholder rubber agroforest system established by planting rubber trees into secondary rainforest^8^. Its implementation dates back to the early 20th century before rubber and oil palm monocultures became more common. The criteria for jungle rubber plot selection was that plots should contain non-rubber trees that are older than rubber and individual rubber trees should not be planted in rows and are of varying ages. All rubber and oil palm plantations were smallholder plantations, meaning that they were owned and managed by small farm households, as opposed to large-scale company plantations. The implicit assumption of our experimental design, comparing the changes in converted land-uses to the reference land use (that is, forest) with assess effects of land-use change, is that the initial soil conditions were comparable before conversion. To test this assumption, we statistically compared land-use independent soil characteristics (that is, soil texture at depths ≥0.5–2 m) among land uses within each landscape. We did not detect significant differences in soil texture between the reference and converted land uses within a soil landscape^30^,^42^, suggesting that soil conditions were previously similar. The measurements in these 32 core plots are described in detail below and summarized in Supplementary Table 5.

### Trees and understorey vegetation

Within each core plot, all trees with a diameter at breast height (DBH) ≥10 cm were identified and measured (height, DBH, crown structure). All vascular plant individuals growing within five 5 × 5 m subplots were identified and measured (height). Whenever possible, herbarium specimens were prepared of three individuals per species for identification and later deposition at several Indonesian herbaria (Herbarium Bogoriense, BIOTROP Herbarium, UNJA Herbarium, Harapan Rainforest Herbarium). To calculate the naturalness of each land-use system, all core plots were surveyed for the presence of the ten most common weed species on plot level (specimens deposited at Herbarium Bogoriense).

### Birds

Birds were sampled with point counts as well as automated sound recordings. The point counts were located in the centre of each plot and all birds within the plot were recorded for 20 min between 6:00 and 10:00 in June–July 2013. The timing of bird data collection alternated between early and late morning and all plots were visited three times. Individuals flying above the canopy were excluded, and unfamiliar bird calls were recorded using a directional microphone. The recordings were compared with the Xeno-Canto online bird call database (http://xeno-canto.org/) for confirmation. In addition, we recorded sound at 44,100 Hz using stereo recorders (SMX-II microphones, SM2+ recorder, Wildlife acoustics) which were attached to the plot's central tree at 2–2.5 m. Eight plots could be sampled simultaneously, so sampling all 32 plots took four days (10th and 13th of May, and the 3rd and 7th of June 2013). The first 20 min from sunset were uploaded to a website (http://soundefforts.uni-goettingen.de/) where two independent ornithologists tagged all audible bird calls (within an estimated 35 m radius) with the corresponding species name. Only bird species identified by both ornithologists in each plot were used and subsequently merged with the species list obtained from the point counts. Finally, each bird's habitat preference was classified based on Beukema *et al*.^44^ to detect forest specialists. Missing bird information was looked up in the online Handbook of the Birds of the World (http://www.hbw.com/).

### Litter invertebrates

Litter macro-invertebrate sampling took place between October and November 2012. In each core plot, we sampled 1 m^2^ in each of three 5 × 5 m subplots. This sampling was done by sieving the complete leaf litter layer from the 1 m^2^ sample through a coarse sieve with a mesh width of 2 cm. A total of 7,472 macro-invertebrates were then hand-collected from the sieved samples and stored in 65% ethanol. Specimens were identified to morphospecies based on consistent morphological characteristics.

### Termites

Termite sampling was conducted in 10 × 50 m transects bisecting each plot. Along each transect, termites were searched for on the soil surface, leaf litter and trees (Fig. 3). Baits made from rubber wood with the volume of 3 × 3 × 50 cm were installed on each of the five 5 × 5 m subplots. Wood baits were inserted into the soil up to half of their length. Baits were harvested after four weeks and the termites collected. Termites obtained from transects and baits were stored in 70% ethanol, labelled, sorted and identified.

### Ants

Ants were collected using direct sampling and baiting. Direct sampling was carried out in three stratum, leaf litter, soil, and tree trunk, and lasted 5–10 min per stratum per subplot. Leaf litter was separated into coarse and fine litter and ants were taken from the fine leaf litter. For the soil and tree strata, ants were collected directly from the ground and trunk with forceps. The baiting method used plastic observation plates with two baits of 2 cm^3^ of tuna and two sponges saturated with 70% sugar solution attached to sample ants. One plate was tied at breast height on each of two trees in each subplot. If there were not two trees in a subplot (often the case in oil palm plantations), the closest trees to the subplot were chosen. The plates were checked at 15, 30, 45 and 60 min after placing the plates on the trees. Specimens were collected from each ant species present where possible without disrupting recruitment. All sampling was completed between 9:00 and 11:00 and never during or immediately after rain due to a reduction in ant activity in wet conditions. Direct sampling was carried out once (February–March 2013), and baiting four times (October–November 2012, February–March 2013, October–November 2013, February–March 2014). All ants collected were identified to species/morphospecies level.

### Testate amoebae (protists)

Samples from the litter/fermentation layer were taken in October–November 2013, using a core of a diameter of 5 cm. Testate amoebae were extracted by washing 1 g litter sample from each core plot over a filter of 500 μm mesh and then back-sieving the filtrate through 10 μm. Microscopic slides were prepared from the final filtrate and testate amoebae were identified to morphospecies.

### Prokaryotic soil community

Soil sampling (top 5–7 cm) was carried out in 2012 for three subplots within each core plot. All samples were stored at −80 °C until further use. Subsequently, deoxyribonucleic acid (DNA) was isolated using the PowerSoil DNA isolation kit (Dianova, Hamburg, Germany). Subsequently, 16S rRNA gene amplicons of bacteria and archaea were generated from DNA. The resulting 16S rRNA gene data sets were processed and analysed using QIIME 1.8 (ref. 45). Initially, sequences shorter than 300 base pairs (bp), containing unresolved nucleotides, exhibiting an average quality score lower than 25, harbouring mismatches longer than 3 bp in the forward primer (Supplementary Data 1), or possessing homopolymers longer than 8 bp, as well as primer sequences, were removed. Subsequently, sequencing noise and potential chimeric sequences were resolved by using Acacia^46^ and UCHIME^47^ with RDP^48^ as reference data set (trainset10_082014_rmdup.fasta). Operational taxonomic unit (OTU) determination was performed at a genetic divergence of 3% by using the software tool pick_open_reference_otus.py of the QIIME 1.8 package using the Silva NR SSU 119 database version as refs 45 and 49. Taxonomic classification was performed with parallel_assign_taxonomy_blast.py against the same database. OTUs representing singletons, chloroplasts, extrinsic domains, and unclassified were removed. OTU tables were subsampled and comparisons were performed at the same surveying effort (Bacteria 6.800 sequences and Archaea 2.000 sequences). Diversity estimates were generated employing alpha_rarefaction.py.

### Genetic diversity of plants

In each of the core plots, ten vascular plant species (woody species and herbaceous plants including ferns), selected based on their dominance in terms of above-ground biomass (AGB), were selected using a modified angle count technique (‘Bitterlich-Method'). From each selected species, leaf material of ten individual plants belonging to the same species was sampled. In total 10 plants/species × 10 species/plot × 32 plots=3,200 plants were sampled. Due to different dominance of species in each plot, a total number of 112 species were sampled. Using the DNeasy 96 Plant kit and its protocol (Qiagen, Hilden, Germany), the total genomic DNA was extracted out of ∼1 cm^2^ dried leaf material. According to the protocol of Vos *et al*.^50^ with minor modifications, all samples were analysed with one AFLP primer combination for all species (Supplementary Data 1). Two samples of each species were repeated from DNA extraction onwards for reproducibility testing. Fragment determination was carried out with the GeneMapper 4.1. (Applied Biosystems). We calculated Shannon Index/genetic diversity with 10 individuals of each species in every plot. Based on the 1–0 matrices, we calculated Shannons information index (I).

### Nutrient leaching fluxes

We installed at random two suction cup lysimeters (P80 ceramic, maximum pore size 1 μm; CeramTec AG, Marktredwitz, Germany) in each of the eight replicate plots of forest, jungle rubber and rubber, and one suction cup lysimeter in each of the eight replicate plots of oil palm. Lysimeters in the oil palm plots were placed at 1.3–1.5 m distance from the palm trunk. In all plots, lysimeters were installed into the soil at 1.5 m depth, which was well below the rooting depth. This was ascertained from the fine and course root distribution with depth, which we measured at 0.1 m depth interval down to 1 m and showed a strong exponential decrease of root mass with depth. Before installation, lysimeters, sample tubes and collection containers were acid-washed and rinsed with copious amounts of deionized water. Lysimeters were installed three months before the first sampling to allow resettling of natural soil conditions before measurement. The collection containers (dark glass bottles) were placed in plastic buckets with lid and buried in the ground far from the lysimeters. Soil water was sampled biweekly to monthly, depending on the frequency of rainfall, from February to December 2013. Soil water was withdrawn by applying 40 kPa vacuum on the sampling tube, which represents soil water in rapidly and slowly draining pores. The collected soil water was transferred into 100 ml plastic bottles and was frozen immediately on arrival at the laboratory. All frozen soil water samples were transported by air to the laboratory of Soil Science Tropical and Subtropical Ecosystems (SSTSE), University of Göttingen, Germany, and remained frozen until analysis. Total dissolved N was determined using continuous flow injection colorimetry (SEAL Analytical AA3, SEAL Analytical GmbH, Norderstedt, Germany). Dissolved organic C was analysed using Total Organic Carbon Analyzer (TOC-Vwp, Shimadzu Europa GmbH, Duisburg, Germany). Dissolved Na, Ca, Mg, total Al, total P and total S were measured using an inductively coupled plasma-atomic emission spectrometer (ICP-AES; iCAP 6300 Duo VIEW ICP Spectrometer, Thermo Fischer Scientific GmbH, Dreieich, Germany). Element concentrations from the two lysimeters per plot were averaged to represent a plot on a sampling period. Leaching flux from each plot was calculated by multiplying the biweekly or monthly element concentrations with the total drainage water flux at 1.5 m depth during two weeks or one month. The drainage water flux was estimated on a daily time step using the soil water module of the Expert-N model^51^, parameterized with climate, leaf area index, rooting depth and soil texture data from our sites. Climate data (daily minimum, maximum and average air temperature, average relative humidity, average wind speed, daily total solar radiation and precipitation) were taken from the meteorological stations of the Indonesian Meteorological, Climatological and Geophysical Agency located at about 10–20 km from our sites for February–June 2013, and for July–December 2013 climate data were measured at a meteorological station installed in each of our two landscapes. Daily evapotranspiration was calculated using Penman–Monteith method, with aerodynamic and canopy conductance adjusted to our sites' conditions. Vegetation data input included leaf area index and root distribution, which were all measured from our plots. Root uptake of water from the soil was depth-partitioned following the measured root-mass distribution with depth. The Richards equation was used to simulate vertical water movement in the soil. The relationships between matrix potential, water content and hydraulic conductivity were derived from the soil texture of our sites. The model was validated by comparing modelled and measured soil matrix potentials. Soil matrix potential was measured in each land-use type monthly using tensiometers (P80 ceramic, maximum pore size 1 μm; CeramTec AG, Marktredwitz, Germany), installed at 0.3 and 0.6 m depths. Finally, the drainage flux was calculated as the net vertical flux at the sampling depth of soil water (1.5 m) and summed for two weeks or one month corresponding to the sampling period. Annual element flux for each plot was then the sum of the biweekly to monthly element fluxes during 2013.

### Soil sampling and fertility characteristics

In each core plot, a 10 × 10 m grid was established and we randomly selected 10 grid points as subplots that were at least 5 m distance from the plot's border for soil sampling^42^. Soil samples were taken within an area of 0.4 × 0.4 m in each grid point. The soil had no organic layer. We removed the thin litter layer to sample predominantly the mineral soil. Soil samples were taken at several depth intervals (0–0.1, 0.1–0.3, 0.3–0.5, 0.5–1.0, 1.0–1.5 and 1.5–2.0 m), and we report here the changes in soil characteristics for the top 0.1 m, except for net N mineralization (which was for the top 0.05 m) and SOC (which was for the entire 2 m). Soil samples were air dried and sieved (2 mm sieve) at the University of Jambi, Indonesia and sent to SSTSE laboratory, University of Göttingen, Germany for analysis. From the air-dried, sieved soil samples, pH was analysed in a 1:4 soil-to-water ratio, and ECEC was determined by percolating the soils with unbuffered 1 mol l^−1^ NH_4_Cl and the percolate cation concentrations (exchangeable bases, Al, Fe and Mn) were measured using ICP-AES. Base saturation was calculated as per cent exchangeable base cations (Ca, Mg, K and Na) of the ECEC. Extractable P was determined using the Bray 2 method, and analysed using ICP-AES. For soil ^15^N natural abundance signature, the 10 sub-samples from each replicate plot was composited, finely ground and analysed using isotope ratio mass spectrometry (Delta Plus, Finnigan MAT, Bremen, Germany). Soil ^15^N natural abundance signature is used as an index of soil N availability because it relates to the gross rates of mineral N production in tropical forest soils^52^. Net N mineralization was measured using the buried bag method on intact soil cores incubated *in situ*; such method excludes N uptake by plants, and thus net N mineralization represents the fraction of mineral N produced in the soil that is available for plant uptake^53^. For this assay, we randomly selected two subplots in each core plot. In each subplot, two intact soil cores were taken in the top 0.05 m depth. One core was extracted immediately in the field with 0.5 mol l^−1^ K_2_SO_4_ solution (*T*_*0*_). The second core was placed in a plastic bag, loosely closed to allow air exchange while preventing rain from entering, inserted back into the soil to incubate *in situ* for 7 days (*T*_*1*_), and then extracted. We used the same mineral N extraction, analytical and calculation methods described in details in our earlier work in tropical ecosystem^54^. SOC was determined from air-dried, sieved and finely ground soil samples taken from the 10 randomly selected subplots per plot for the top 0.5 m depth and from the two subplots per plot for the depths ≥0.5–2.0 m (see above). SOC was analysed using a CN analyzer (Vario EL Cube, Elementar Analysis Systems GmbH, Hanau, Germany). SOC stock for each depth interval was calculated from the SOC concentration and the measured bulk density, as described by de Blécourt *et al*.^55^. Soil bulk density was measured at the same depth intervals, using the soil core method^56^ in soil pits dug right beside the core plots. Total SOC stocks down to 2 m depth were calculated as the sum overall depth intervals. Land-use changes often coincide with changes in bulk density due to management practices, which may compact or loosen the soil. To be able to compare the same soil mass and to avoid the interference of bulk density changes with SOC stock changes, we used the bulk density of the reference land-use (that is, forest) to calculate the SOC stock of the converted plantations^57^. Statistical analyses for all soil fertility parameters were conducted on the average values of the subplots that represent each plot, and thus *n*=32 plots across land uses and landscapes.

### Soil processes and functioning

Litterbags (20 × 20 cm with 4 mm mesh size), containing 10 g dry leaf litter mixture of three tree species from one of the forest plots, were incubated *in situ* with one litterbag in each of the 32 plots from October 2013 to March 2014. The composition of the litter reflected that of fallen litter at the plot of origin: 4 g from cf. *Garcinia* sp., 3 g from *Gironniera nervosa*, 3 g from cf. *Santiria lavigata*. Mass loss was calculated as the difference between the initial litter dry mass and litter dry mass remaining after 6 months and expressed as percentage of the initial leaf litter mass. In addition, soil samples down to a depth of 10 cm were taken with a corer (5 cm diameter) at three subplots in each of the 32 plots. From these soil samples, basal respiration and microbial biomass were determined by measuring O_2_ consumption using an automated respirometer system^58^. Microbial specific respiration was calculated as μl O_2_ mg^−1^ C_mic_ h^−1^.

### Stability in climatic conditions

To evaluate the stability of climatic conditions, weather stations were installed in the centre of the 32 core plots. They were equipped with thermohygrometers (Galltec Mella, Bondorf, Germany) placed at a height of 2 m above the ground to record air temperature and humidity inside the canopy, and soil sensors (IMKO Trime-PICO, Ettlingen, Germany) at a depth of 0.3 m to monitor soil temperature and moisture. Both sensors were connected to a data logger (LogTrans16-GPRS, UIT, Dresden, Germany) and measurements were taken every hour. Ranges and percentiles of 5 and 95% were calculated for all variables for the period of June 2013–October 2014.

### Above- and below-ground biomass and carbon stock

In each core plot, all trees, palms and lianas with DBH>10 cm were tagged, the DBH at 1.3 m tree height was measured with a measuring tape (Richter Measuring Tools, Speichersdorf, Germany), and tree height was recorded using a Vertex III height meter (Haglöf, Långsele, Sweden). Wood density values (dry mass per fresh wood volume in g cm^−3^) were determined in extracted wood cores of 204 trees and interpolated values were applied for remaining trees based on a calibration equation with pin penetration depth measured with Pilodyn 6J wood tester (PROCEQ SA, Zürich, Switzerland). Understory trees in forest plots with a DBH of 2–9.9 cm were inventoried in the same way on two 5 × 5 m subplots in each plot. To convert the recorded tree structural data into AGB, we applied the allometric equations for forest trees^59^, rubber trees^60^, oil palms^61^ and lianas^62^. Coarse root (>2 mm diameter) and its biomass were estimated using allometric equations for forest trees^63^, rubber trees^60^ and oil palm^64^. We added our measurements of small-diameter (≤2 mm) root biomass to the estimated coarse root biomass. Fine-root biomass was measured using 10 soil cores (3.5 cm in diameter) from the top down to 50 cm soil depth on each plot. All fine-root segments longer than 1 cm were extracted by washing over a sieve of 200 μm mesh size (Retsch, Haan, Germany) and separated under a stereomicroscope into live (biomass) and dead fractions (necromass). Woody coarse debris was analysed within all forest and jungle rubber plots, where snags (DBH >10 cm) and logs (mid-point diameter >10 cm, length >1 m) were recorded. Three decay stages based on from Grove^65^ were used to characterize the woody debris, and debris mass was calculated using the equations by Kauffmann and Donato^66^ and by applying the allometric equation by Chave *et al*.^59^ for calculation of AGB of un-degraded trees.

### Net primary productivity

We measured above-ground litterfall, pruned oil palm fronds, rubber latex harvest, oil palm fruit harvest, and stem increment from March 2013 to April 2014. For litter collection, 16 litter traps (75 × 75 cm), made from polyvinyl chloride tube frames and nylon mesh (mesh size of 3 mm) and mounted on 1 m-long wooden stakes, were placed on each of the plots except in oil palm plantations (*n*=24) in randomly selected grids in each plot. Litter collection was done at monthly intervals and the collected litter was sorted into leaves, small woody material (diameter <2 cm), propagules and inflorescences, which were subsequently oven-dried for 72 h at 60°C until constant mass was attained. In the oil palm plantations, all pruned oil palm fronds on each plots were counted. The average dry weight per frond, obtained from 16 harvested and dried fronds, was used for the calculation of litter production. Oil palm was harvested every two weeks, while rubber was harvested at frequencies depending on season and expected productivity. The yield of oil palm fruits and rubber latex (in Mg ha^−1^) was recorded by weighing the harvested fresh material for all trees in each plot. The dry weight was then determined after oven-drying representative sub-samples of oil palm fruits (five multiple fruits) and rubber latex (five harvest bowls) at 70 °C to constant mass. From these data and the area of the plots we obtained the yield as dry weight per hectare. Annual above-ground tree woody biomass production (Mg ha^−1^yr^−1^) was calculated from stem increment, measured with dendrometer (UMS, München, Germany), of 40 trees per plot (960 trees in total). The cumulative biomass increment of each tree was calculated as the mass difference of a tree between March 2013 and April 2014, based on the allometric equations used for biomass estimation as described above. For a plot-based estimation of above-ground tree biomass, we applied mean increment rates per plot and tree species for the remaining tree individuals. An in-growth core measurement was conducted to estimate fine-root productivity in all plots, using the method described by Powell and Day^67^. Sixteen in-growth cores per plot were installed at random locations (at 30 cm distance from the litter traps) and re-sampling of the cores was done after 8–10 months. The extracted soil cores were processed in the same manner as done for the fine-root inventory. The fine-root growth in the cores was extrapolated to one year and expressed in g dry mass produced per m^2^ surface area per year, representing the annual fine-root production. To determine carbon stocks and carbon sequestration, the C concentration of stem wood, fine roots, dead wood, and litter fractions was analysed with a CN Analyzer (Vario EL III, Hanau, Germany) at the University of Göttingen. For all methodological details see Kotowska *et al*.^68^.

### Statistical methods

Data were standardized by subtracting the mean and dividing by the standard deviation. Species richness, nutrient flux and yield data were log_10_-transformed before standardization to avoid heteroscedasticity. When a single response was indicated by a single measured indicator variable, we used general linear models for ecological data, and linear mixed models with village as a random grouping factor for household-based data. When a single response was indicated by multiple measured indicator variables, we used linear mixed models with plot (ecological data), or household nested in village (household data), as a grouping variable. In addition, when including an indicator variable expected to negatively affect the response, we used its additive inverse (see soil fertility and stability in climatic conditions). The significance of the transformation system effect and its interaction with the identity of the indicator variables was assessed using F/Wald-tests on models fitted using Maximum-likelihood. For the household data we also included these tests for the type of village (transmigrant vs. non-transmigrant) and its interaction with transformation system. Differences among transformation systems are assessed using Tukey post-hoc tests. Analyses were done in R 3.1.2 (ref. 69) with packages ‘nlme' v.3.1-118 (ref. 70) and ‘multcomp' v.1.3-8 (ref. 71).

### Data availability

Data is archived at EFForTs-IS^72^, with openly accessible, keyword-searchable metadata and data holder contact details for data requests. Datasets used in this study have the identification numbers 11250, 11253, 11254 and 11257 (household survey), 11441 and 11460 (attitudes to risk), 12027, 12028, 12029 and 12030 (land-use/land cover), 11161 (soil processes), 12104 (leaching), 11481, 11482 and 11484 (yields), 12002 (biomass and productivity), 11485 (tree structure), 12221 and 12200 (plant genetic diversity), 11922, 11923, 11924 and 11925 (plant diversity), 11720, 11725 and 13061 (birds), 11780, 11966, 12266 and 12942 (macro-invertebrates), 12180, 12220, 12344, 12341, 12342 and 12343 (invasive plants), 12322 (decomposition), 12321 (testate amoebae), 12013 (microbial biomass and basal respiration) 11742 (16S rRNA gene based analysis of soil archaeal communities, DNA), 11740 and 12264 (16S rRNA gene based analysis of soil bacterial communities, DNA), 11660 (meteorological data). For the prokaryote data, 16S rRNA gene sequences were deposited in the National Center for Biotechnology Information (NCBI) Sequence Read Archive (SRA) under study accession number SRP056374.

## Additional information

**How to cite this article:** Clough, Y. *et al*. Land-use choices follow profitability at the expense of ecological functions in Indonesian smallholder landscapes. *Nat. Commun.* **7,** 13137 doi: 10.1038/ncomms13137 (2016).

## Supplementary Material

Supplementary Information Supplementary Figures 1 - 9, Supplementary Tables 1 - 5, Supplementary Note 1 and Supplementary References

Supplementary Data 1 Primer pair sequences for plants, bacteria, and archaea

## Figures and Tables

**Table 1 t1:** Land-use/land cover change (%) from 1990 to 2011 in the study landscapes in Jambi Province (Indonesia) based on land-use classification inferred from remote sensing.

**Land-use/Land Cover**	**2011**					**Total 1990**	**Loss**
	**Forest**	**Oil palm**	**Other**	**Rubber**	**Shrub/bush**		
1990							
Forest	36.60	10.59	0.89	5.79	12.66	66.52	29.92
Oil palm		7.46	0.01	0.00	0.06	7.53	0.06
Other		0.66	1.10	0.06	0.32	2.14	1.04
Rubber		0.03	0.01	20.39	0.00	20.43	0.04
Shrub/bush		0.04	0.00	0.00	3.35	3.38	0.04
Total 2011	36.60	18.77	2.00	26.24	16.39	100.00	
Gain	0.00	11.31	0.90	5.85	13.04		

**Table 2:** Statistical results including interactions between indicator variable and land-use system.

**Response variable**	**Explanatory variable**	**numDF**	**denDF**	**Wald/*****F*****-value**	***P*** **value**
Material inputs	Land-use system	1	955	45.41	<0.0001
	Variable	2	955	0.00	1.0000
	Village type	1	955	3.40	0.0655
	Interaction LUS × Var	2	955	11.56	<0.0001
	Interaction LUS × VT	1	955	1.77	0.1713
	Interaction Var × VT	2	955	11.42	<0.0001
	Interaction LUS × VT × Var	2	955	3.77	0.0048
Labour inputs	Land-use system	2	496	83.24	<0.0001
	Variable	1	496	0.00	1.0000
	Village type	1	496	0.68	0.40
	Interaction LUS × Var	2	496	10.08	0.0001
	Interaction LUS × VT	2	496	0.37	0.6942
	Interaction Var × VT	1	496	0.07	0.0737
	Interaction LUS × VT × Var	2	496	6.73	0.0013
Gross margin per ha	Land-use system	2	414	58.55	<0.0001
	Village type	1	414	10.97	0.0010
	Interaction	2	414	0.28	0.7533
Gross margin per labour hour	Land-use system	2	416	16.74	<0.0001
	Land-use system	2	414	7.01	0.0084
	Village type	1	414	0.82	0.4429
Holt–Laury	Land-use system	2	84	3.27	0.0427
Naturalness	Land-use system	3	29	95.55	<0.0001
	Variable	2	54	0.95	0.9676
	Interaction	6	54	2.27	0.0502
Biodiversity	Land-use system	3	199	10.29	0.0001
	Variable	8	199	0.03	1.0000
	Interaction	24	199	6.58	<0.0001
Genetic plant diversity	Land-use system	3	28	7.60	<0.0001
Stability in climatic conditions	Land-use system	3	28	22.93	<0.0001
	Variable	4	112	0.00	1.0000
	Interaction	12	112	7.81	<0.0001
Soil processes and functioning	Land-use system	3	28	6.81	0.0014
	Variable	3	84	0.00	1.0000
	Interaction	9	84	1.43	0.1871
Nutrient leaching fluxes	Land-use system	3	26	7.11	0.0012
	Variable	7	182	0.00	1.0000
	Interaction	21	182	1.73	0.0291
Yield	Land-use system	2	21	74.94	<0.0001
NPP	Land-use system	3	28	34.81	<0.0001
Carbon stocks	Land-use system	3	31	11.61	<0.0001
	Variable	1	25	0.00	1.0000
	Interaction	3	25	6.85	0.0016
Soil fertility	Land-use system	3	26	7.72	0.0008
	Variable	5	130	0.00	1.0000
	Interaction	15	130	2.10	0.0132

The multiple indicator variables used for each response variable are shown in Figure 4; because indicator variables may systematically differ in responses to land use, we test the interaction between indicator variable identity (listed in the table as Variable) and land-use. Linear models and *F*-tests were used with models with a single indicator variable, linear mixed models and Wald-tests for models with multiple indicator variables. denDF, denominator d.f. for *F*-tests; LUS, land-use system; NPP, net primary production; numDF, numerator d.f. for *F*-tests; Var, variable; VT, village type.
