# Supplementary material for: Land-use choices follow profitability at the expense of ecological functions in Indonesian smallholder landscapes
Source: Nat Commun. 2016 Oct 11;7:13137. doi: 10.1038/ncomms13137 (PMC5062595; doi:10.1038/ncomms13137)
Supplement: Supplementary Information — Supplementary Figures 1 - 9, Supplementary Tables 1 - 5, Supplementary Note 1 and Supplementary References [file ncomms13137-s1.pdf]

Supplementary Figures

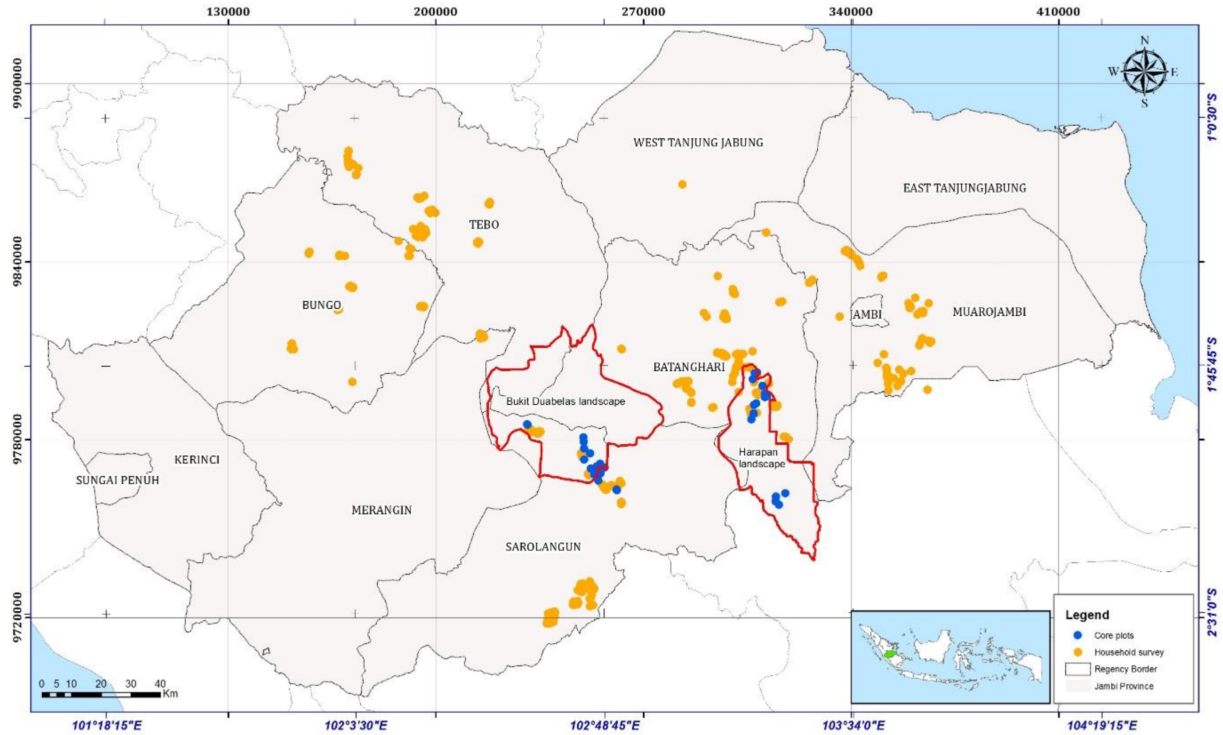

Supplementary Fig. 1. Map of the study area with villages sampled for households (orange) and the landscapes (red) with the core plot sites (blue) highlighted. The figure was produced by taking as a basis the official Regency Border map from Peta Rupa Bumi Indonesia (Indonesian Geospatial Information Agency) and Badan Pusat Statistik Indonesia (Statistics Indonesia). The location of core plots (blue dots) and household surveys (orange dots) were determined with a hand-held GPS with accuracy <5m. The red polygons show the surroundings of the two main remaining tropical lowland rainforest in the Jambi province: Harapan Rainforest and Bukit Duabelas National Park. These two areas were the reference for the analysis of land use change.

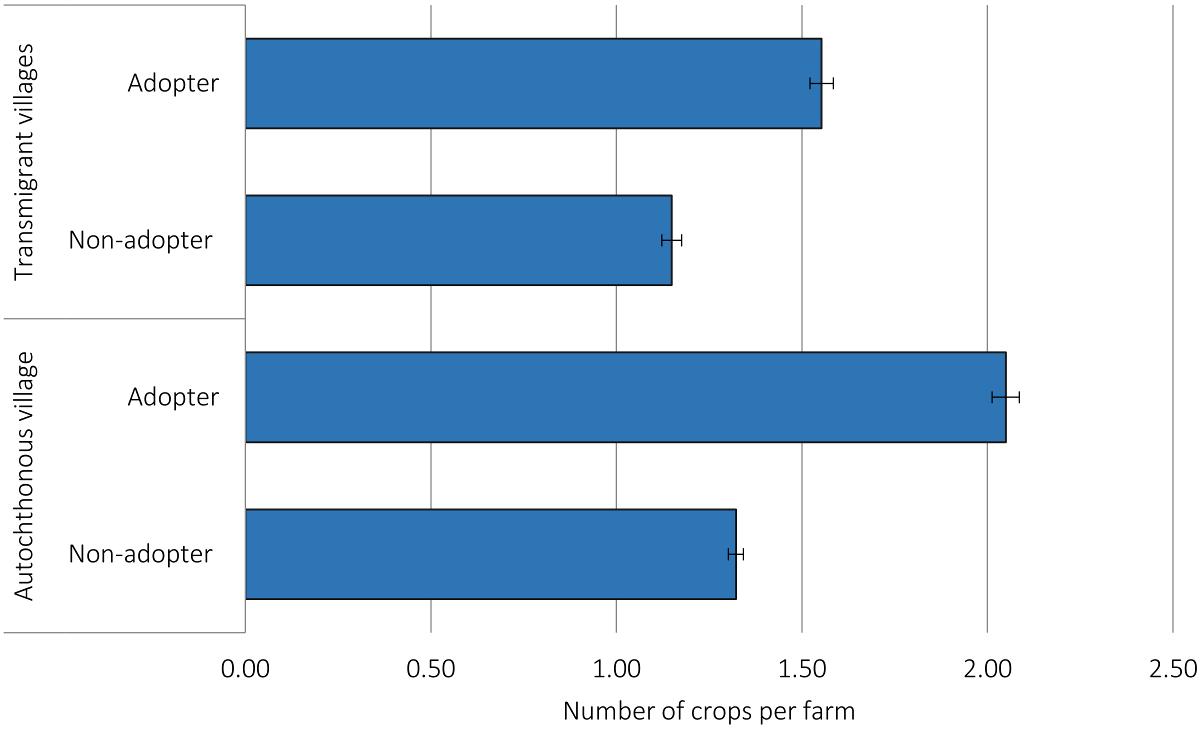

Supplementary Fig. 2. Average number of crops cultivated by households in Jambi, Indonesia. The error bars represent standard error of sample mean values. Adopter and non-adopter categories represent the adoption status with respect to oil palm.

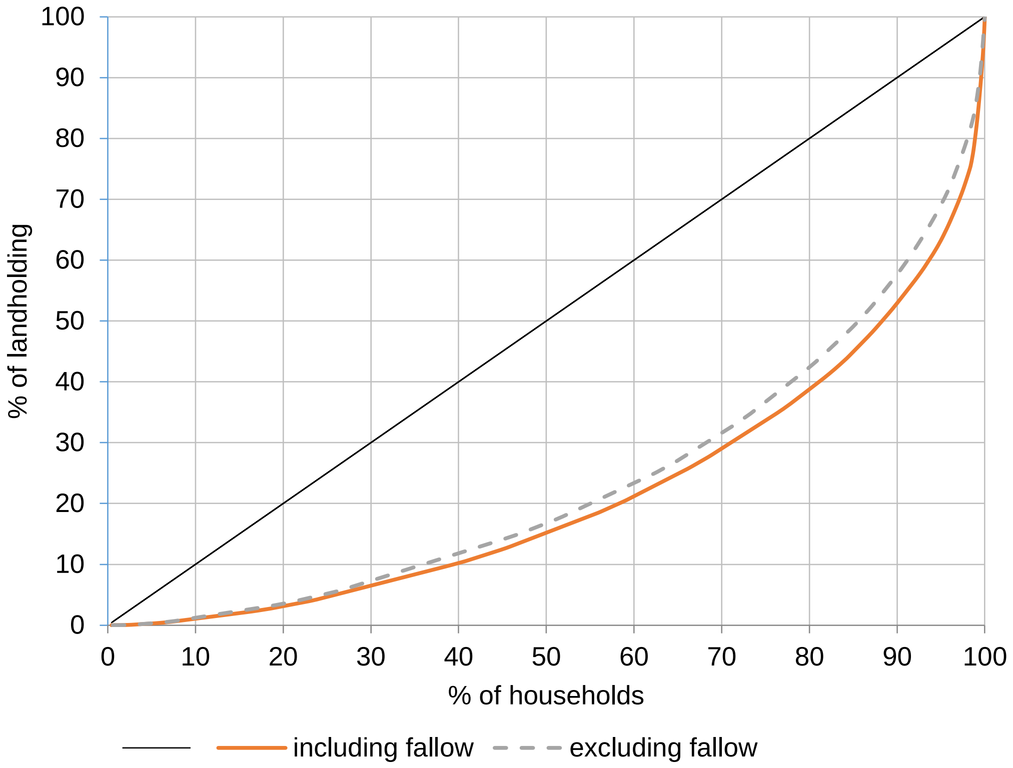

Supplementary Fig. 3. Inequality of landholding distribution among the sample households. Cumulative landholding size was estimated at each data point after arranging the sample households in the increasing order of landholding size, and percentage share was calculated by dividing it with the aggregate landholding size of all households in the sample. When plotted against the percentage of households, the resulting graph would represent the extent of inequality within the sample with respect to landholding size. There wouldn’t be any inequality in the distribution if all households were having landholdings of equal size (uniform distribution with zero inequality, which is represented by the straight line). The curved lines are the Lorenz curves (including and excluding fallow land in the landholding). Inequality of a distribution is measured as the ratio of area between the Lorenz curve of the distribution and the uniform distribution line, and the area under the uniform distribution line (1).

A

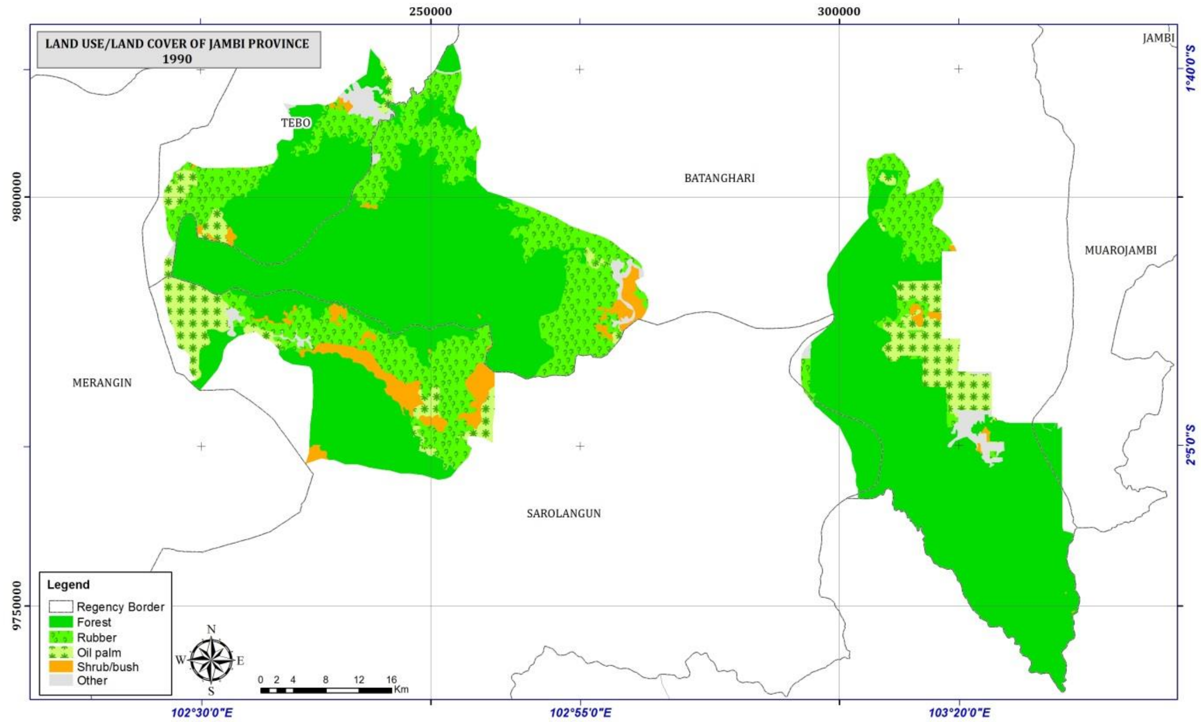

B

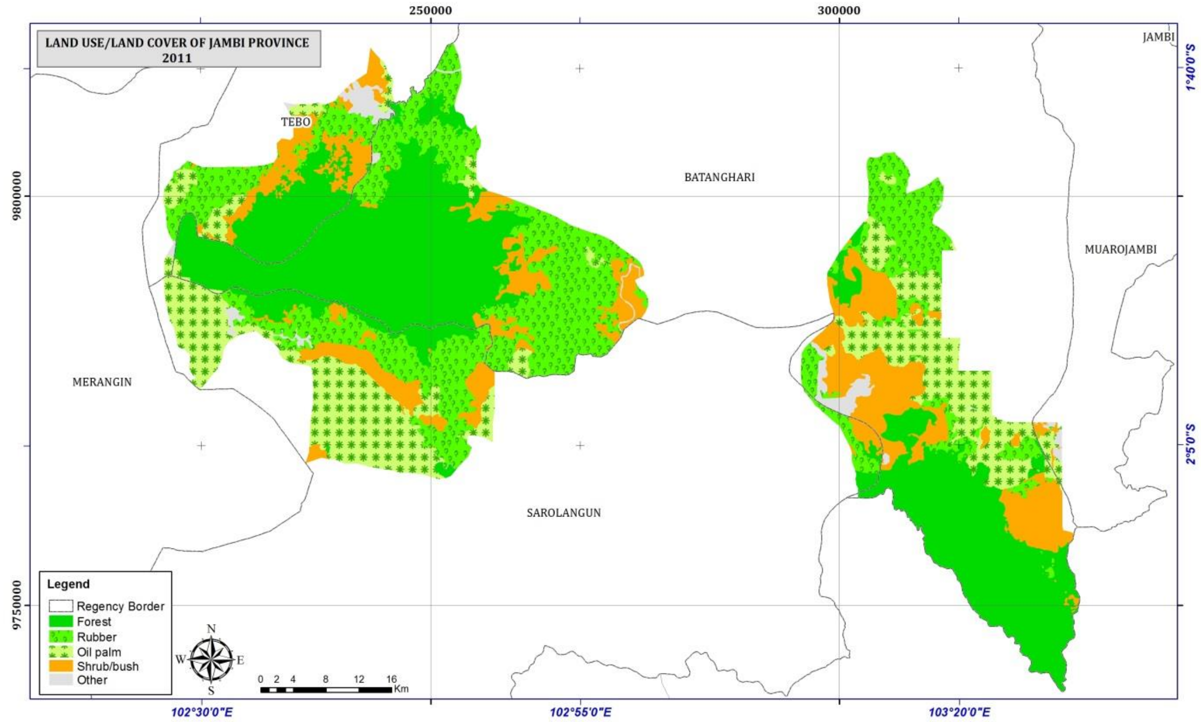

Supplementary Fig. 4. Maps of 1990 (A) and 2011 (B) land use/land cover for the study landscapes.

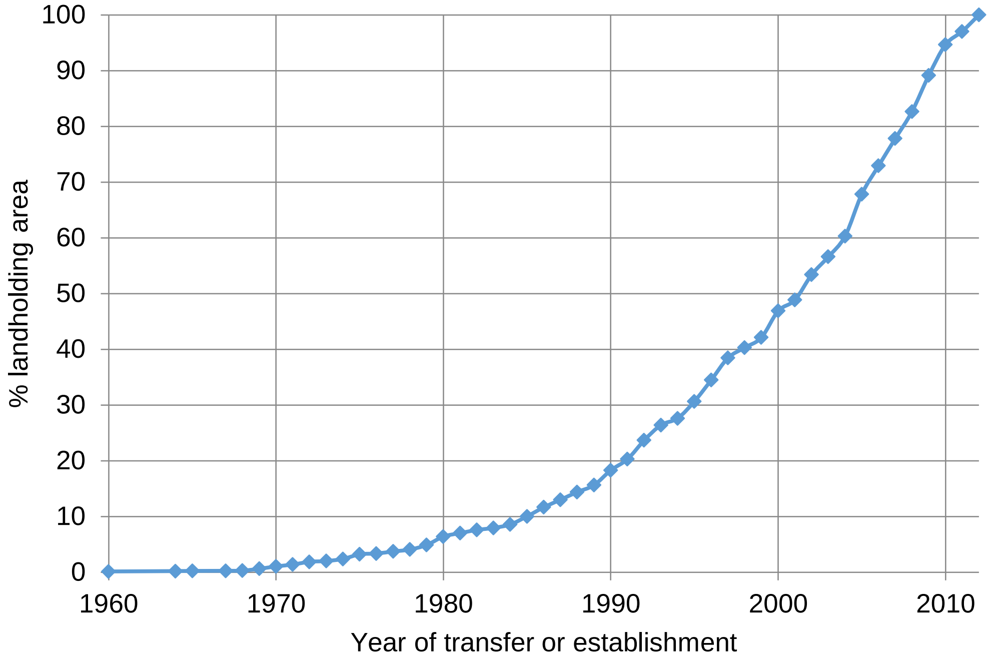

Supplementary Figure 5. Time of establishment of current plantation plots.

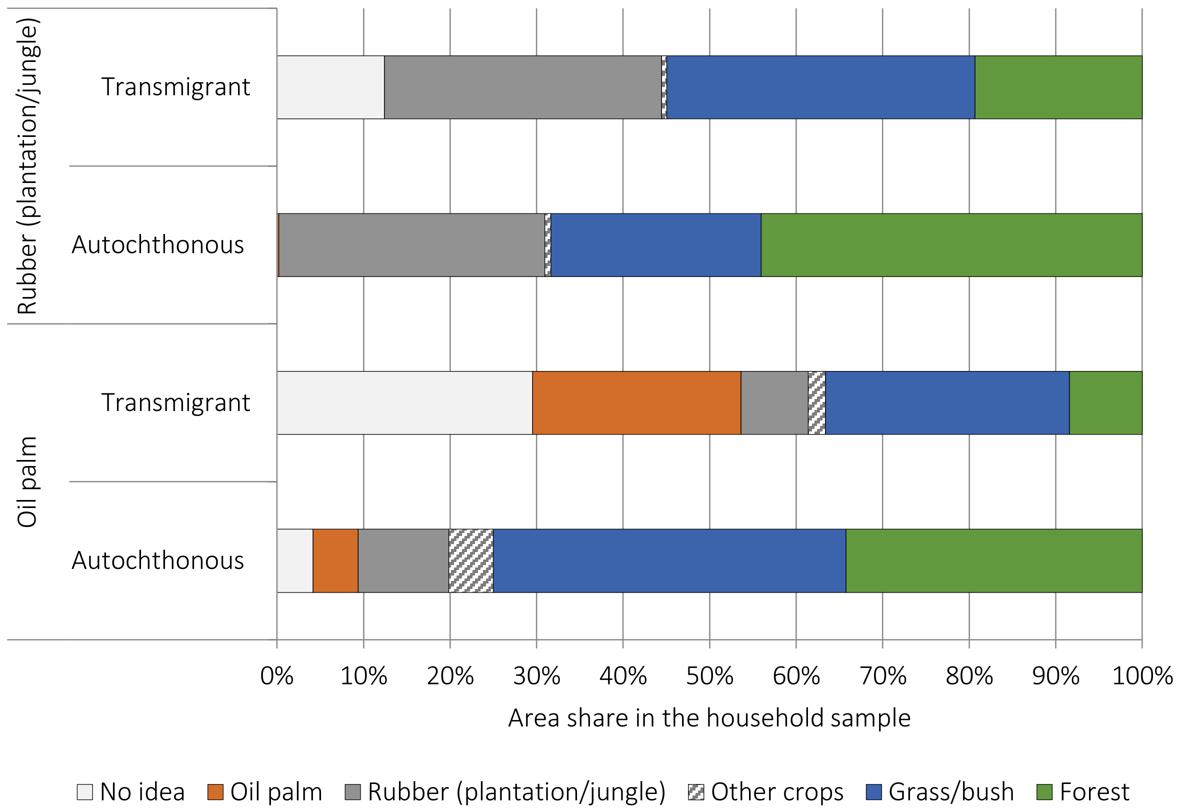

Supplementary Fig. 6. Land-use trajectories with respect to village and crop types. Percentage of area coverage in the sample households is indicated by the width of the colour bars, with land-use type indicated in the legend.

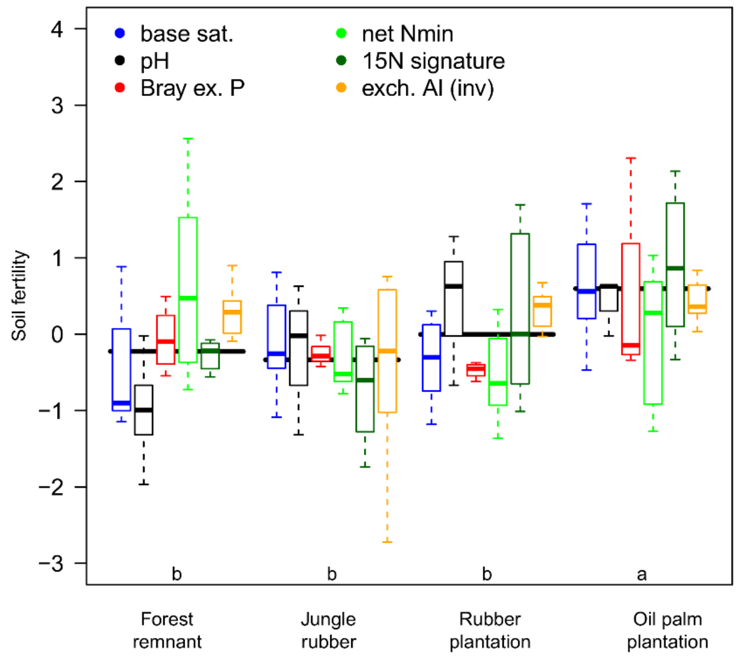

Supplementary Fig. 7. Indicators for soil fertility as a result of management history and recent fertilization in four land-use types in mosaic agricultural landscapes (Jambi, Indonesia). Indicators for soil fertility are: base saturation, pH, Bray-extractable P, net N mineralization, soil ^{15}N natural abundance and exchangeable aluminum (additive inverse). All variables were standardized; summary statistics of raw variables are in Table S3. Horizontal bars indicate the estimated mean indicator variable value; letters indicate significantly different groups.

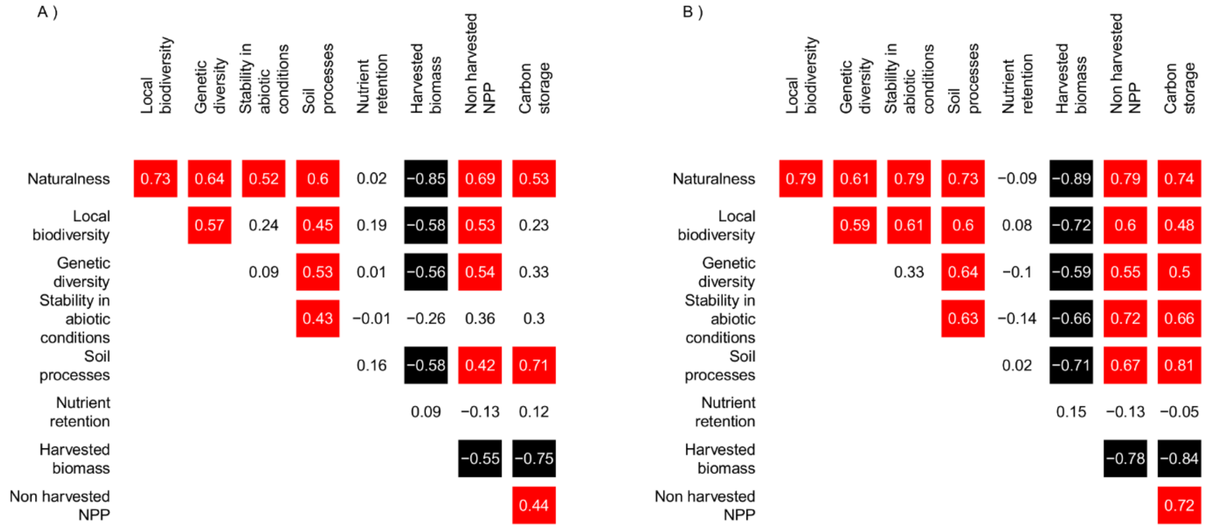

Supplementary Fig. 8. Correlations (Pearson r values) between ecological functions. Correlations are given for (A) agricultural land-use systems (jungle rubber, rubber plantations and oil palm plantations) and (B) across all systems (including forest remnants). Black and red colours refer to significant negative and positive correlations, respectively ($\alpha<0.05$, two-sided test).

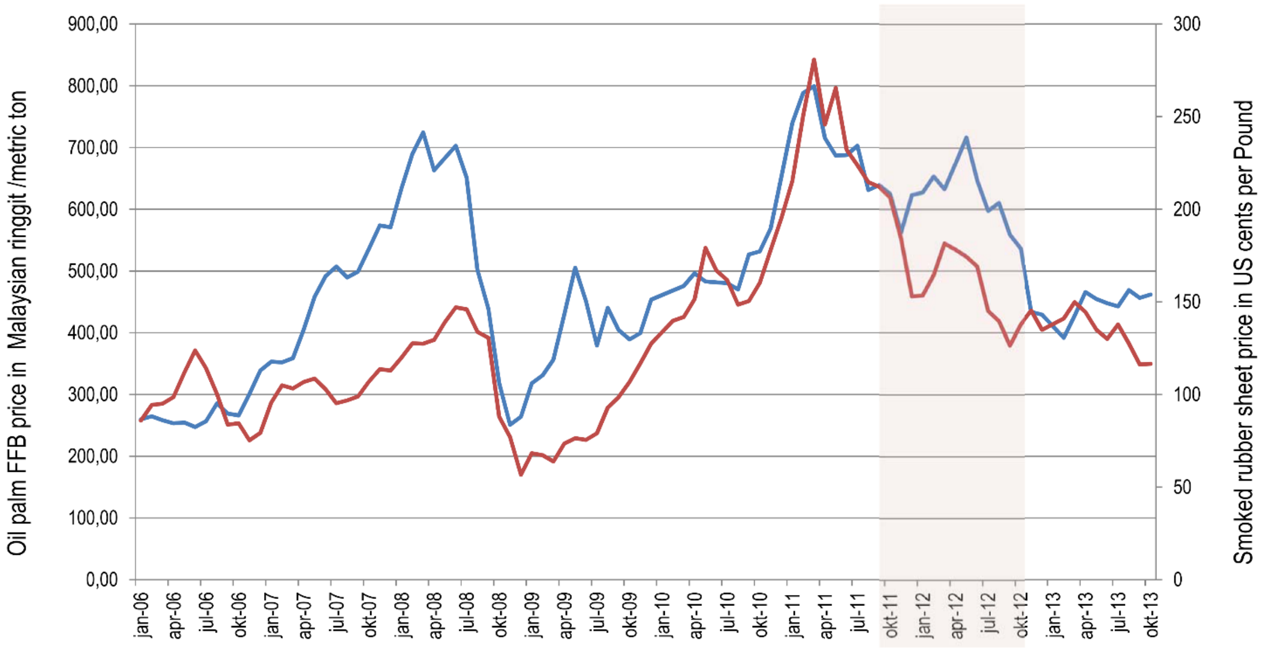

Supplementary Fig. 9. International price movement for oil palm (red) and rubber (blue). Shaded area shows the period covered during the household survey. Data retrieved from http://www.indexmundi.com/, which has used the World Bank Global Economic Monitor (GEM) Commodity database.

Supplementary tables

Supplementary Table 1. Prevalence of crops at the time of household survey (2012).

Crops	Percentage of occurrence in the farm-households (%)	Average area (ha/household)*
Oil palm	35.10	3.58
		(0.37)
Rubber	82.31	3.71
		(0.23)
Other perennials	6.28	0.89
		(0.15)
Annuals	11.70	0.48
		(0.06)
Fallow land	22.83	4.59
		(1.16)

* Conditional on possession. Figures in parentheses show standard errors.

Supplementary Table 2. Mean, standard deviation and number of observations for the untransformed, unscaled indicators for inputs and gross margins for the four land-use systems, for the whole dataset and split up across transmigration and non-transmigration villages. 1 US$ = 9,387 IDR in 2012 (World Bank online database, 2015).

	Jungle Rubber			Rubber plantation			Oil palm plantation		
	mean	sd	N	mean	sd	N	mean	sd	N
Fertilizer cost per ha (10^3 IDR ha^{-1} yr^{-1})	378	823	33	241	548	162	1862	1910	270
Transmigration villages	604	1318	6	523	749	45	2395	2189	137
Non-transmigr. villages	328	696	27	133	402	117	1299	1374	133
Herbicide cost per ha (10^3 IDR ha^{-1} yr^{-1})	124	191	33	134	213	162	286	269	270
Transmigration villages	207	280	6	204	243	45	279	254	137
Non-transmigr. villages	107	168	27	107	196	117	293	284	133
Other input costs per ha (10^3 IDR ha^{-1} yr^{-1})	153	397	33	211	453	162	267	672	270
Transmigration villages	600	828	6	153	300	45	219	598	137
Non-transmigr. villages	53	78	27	233	517	117	316	738	133
Family work hours per ha	505	506	33	765	817	162	107	121	270
Transmigration villages	1143	597	6	887	645	45	116	108	137
Non-transmigr. villages	363	365	27	718	872	117	102	140	133
Hired work hours per ha	201	306	33	278	517	162	97	126	270
Transmigration villages	0	0	6	232	574	45	105	140	137
Non-transmigr. villages	246	322	27	296	495	117	88	110	133
Gross margin per area (10^3 IDR ha^{-1} yr^{-1})	11496	6351	33	18417	11211	162	10534	8547	269
Transmigration villages	15849	8111	6	21628	8598	45	13898	9015	136
Non-transmigr. villages	10529	5627	27	17181	11869	117	7077	6402	133
Gross margin per labour hour (10^3 IDR h^{-1})	21.28	19	33	22.30	17	162	61.81	62	269
Transmigration villages	14.78	7.57	6	25.97	17.97	45	78.97	72	136
Non-transmigr. villages	22.76	21.05	27	20.89	16.95	117	44.62	43	133

Supplementary Table 3. Mean, standard deviation and number of observations for the untransformed, unscaled indicators for ecological functions and services for the four land-use systems

	Forest			Jungle Rubber			Rubber plantation			Oil palm plantation		
	mean	sd	N	mean	sd	N	mean	sd	N	mean	sd	N
Naturalness												
forest bird species (% of total)	11.4	4.3	8	5.4	1.7	8	3.5	1.4	8	0.3	1.5	7
Indigenous trees (% of total)	75.3	17.4	8	36.4	13.3	8	0.5	0.8	8	0.3	0.7	8
Weeds (number of common invasive species absent)	9.0	1.4	8	6.5	1.3	8	3.4	1.7	8	1.2	1.4	8
Local biodiversity (N species/ OTUs)												
Trees	75.2	17.4	8	37.4	13.2	8	1.5	0.8	8	1.3	0.7	8
understory plants	209.4	52.0	8	115.2	24.9	8	47.5	14.6	8	51	15.8	8
Birds	14.0	5.3	8	10.0	3.1	8	10.4	4.8	8	6.3.0	1.4	7
litter inv.	97.0	22.7	8	83.6	19.0	8	76.6	31.2	8	52.2	22.2	8
Termites	11.0	1.4	2	13.0	2.2	4	7.0	0.0	4	4.0	1.8	4
Ants	25.1	5.4	8	22.6	6.3	7	26.1	4.7	8	27.1	6.1	7
testate amoebae	32.1	14.8	8	30.5	17.0	8	14.1	5.0	8	21.6	10.0	8
archaea	123.9	28.1	7	113.0	46.7	8	105.9	43.7	8	114.6	23.2	8
bacteria	957.1	166.5	8	1052.1	127.0	8	1258.8	121.7	8	1373.9	177.5	8
Genetic diversity of dominant plant species												
Shannon Index	0.285	0.035	8	0.291	0.028	8	0.235	0.014	8	0.230	0.046 0	8
Stability in abiotic cond.												
Air Temp. 95th percentile	28.3	0.4	8	30.2	0.5	8	31.1	0.3	8	31.2	0.5	8
Humidity 5th percentile	82.7	4.8	8	71.0	3.2	8	64.5	2.7	8	66.9	2.6	8

Air temp. range	6.3	0.4	8	8.4	0.5	8	9.3	0.3	8	9.4	0.5	8
Soil temp. range	1.3	0.5	8	1.2	0.5	8	1.6	0.4	8	1.7	0.6	8
Soil moisture range	7.7	2.4	8	7.6	3.2	8	7.1	3.3	8	5.5	1.4	8
Soil processes and functioning												
Soil basal respiration (µg O_2*h^{-1} *g soil dw^{-1})	6.3	1.5	8	5.9	1.8	8	4.1	1.0	8	4.5	2.0	8
Soil microbial biomass (µg C_{mic}*g soil dw^{-1})	456.4	115.7	8	529.0	169.0	8	414.8	104.9	8	406.6	152.9	8
Soil specific respiration (µl O_2 mg^{-1} C_{mic} h^{-1})	14.0	2.3	8	11.8	2.2	8	10.7	2.1	8	11.1	2.9	8
Litter mass loss after 6 months (%)	35.6	12.0	8	27.3	14.9	8	13.5	6.5	8	10.6	5.7	8
Nutrient leaching fluxes (1.5-m depth; kg ha^{-1} yr^{-1})												
NH_4^+-N	2.2	0.8	8	3.4	4.2	8	1.9	0.2	8	2.6	0.7	6
NO_3^--N	1.2	1.2	8	0.8	1.0	8	1.8	3.1	8	8.7	13.0	6
Total dissolved nitrogen	4.7	2.0	8	5.3	5.0	8	4.6	2.9	8	12.6	12.8	6
DOC	37.7	9.3	8	58.0	21.9	8	37.4	3.8	8	67.7	16.4	6
Na	31.3	9.6	8	34.6	11.1	8	28.1	4.8	8	96.6	93.1	6
Ca	8.4	2.1	8	10.3	4.6	8	8.9	1.8	8	28.3	24.8	6
Mg	3.3	1.0	8	3.8	1.8	8	3.8	1.4	8	7.2	3.2	6
Total Al	3.1	1.7	8	2.7	1.6	8	3.7	1.0	8	12.9	18.5	6
Total P	0.1	0.1	8	0.1	0.0	8	0.0	0.0	8	0.1	0.0	6
Total S	1.9	0.6	8	1.9	1.4	8	1.3	0.1	8	2.1	0.5	6
Harvested biomass (kg per plot)	-	-	-	832	642	8	31683	9313	8	3740	2402	8
Net primary productivity (Mg ha^{-1} yr^{-1}, without yield)	11.6	1.8	8	9.2	1.3	8	5.7	0.9	8	6.3	1.0	8
Carbon stocks												
Carbon total	195.2	36.7	8	77.5	9.2	8	38.3	11.4	8	27.8	5.9	8

biomass (Mg ha^{-1})												
Soil organic carbon (Mg C m^{-2} within 2-m depth)	208.3	69.9	8	221.8	68.3	8	196.3	69.2	8	176.8	53.3	8
Soil fertility (within 0.1-m depth)												
Base saturation (%)	16.8	9.9	8	21.4	8.1	7	20.6	10.4	8	32.3	13.8	7
pH	4.2	0.1	8	4.4	0.1	7	4.5	0.2	8	4.5	0.1	7
Bray extractable P (mg P m^{-2})	941.0	507.0	8	700.3	190.6	7	449.6	202.0	8	2120.4	2443.5	7
Net N mineralization (mg N kg^{-1} day^{-1})	1.0	0.5	8	0.6	0.2	7	0.6	0.5	8	0.7	0.4	7
^{15}N natural abundance (‰)	4.4	0.3	8	4.2	0.5	7	4.8	0.7	8	5.3	0.6	8
Exchangeable Al (x10^{3}; mg Al m^{-2})	42.03	31.59	8	52.55	36.17	7	38.96	25.3	8	28.51	7.75	7

Supplementary Table 4. Relevant permits, recommended by the Indonesian Institute of Sciences LIPI and issued by the Forestry authorities, which concern the work in the present manuscript

Researchers	Permit numbers
Kevin Darras, Lisa H. Denmead , Damayanti Buchori, Idham Harahap, Teja Tscharntke, Yann Clough, Akhmad Rizali, Walesa Edho Prabowo, Ratna Rubiana	Research permits:<br>211/SIP/FRP/SM/VI/2012<br>Export permits:<br>S.11/KKH-2/2013<br>S.591/KKH-2/2014<br>Collection permits:<br>11055/IT3/PL/2012<br>Collection permit and Transfer of collection permit (SAT-DN) from LIPI:<br>2700/IPH.1/KS.02/XI/2012.
Marife D. Corre, Edzo Veldkamp, Kara Allen, Syahrul Kurniawan, Aiyen Tjoa	Research permits:<br>215/SIP/FRP/SM/VI/2012<br>44/EXT/SIP/FRP/SM/V/2013<br>Collection permits:<br>2703/IPH.1/KS.02/XI/2012<br>S.13/KKH-2/2013
Natalie Breidenbach, Reiner Finkeldey	Research permits:<br>256/SIP/FRP/SMVII/2012,<br>24/EXT/SIP/FRP/SM/III/2013<br>2839/FRP/SM/IX/2014<br>Collection permits:<br>2697/IPH.1/KS.02/XI/2012<br>493/IPH.1/KS.02.04/II/2015<br>Export permits:<br>S.09/KKH-2/2013<br>S.471/KKH-2/2014<br>76/KKH-5/TRP/2013<br>17/KKH-5/TRP/2015
Ulrich Brose, Malte Jochum, Andrew David Barnes	Research permits:<br>338/SIP/FRP/SM/VI/2012 (ADB)<br>s203/SIP/FRP/SM/VI/2012 (MJ)<br>Export permit:<br>51/KKH-5/TRP/2014<br>Collection permit:<br>2695/IPH.1/KS.02/XI/2012
Rolf Daniel, Dominik Schneider	Collection and export permits:<br>S.08/KKH02/2013<br>152/KKH-5/TRP/2013
Elvira Hörandl, Nicole Opfermann, Sri Sudarmiyati Tjitrosoedirdjo	Research permit (Nicole Opfermann):<br>129/SIP/FRP/SM/IV/2013;<br>Collection permit:<br>1243/IPH.1/KS.02/V/2013;<br>Export permits:<br>162/KKH-5/TRP/2013<br>116/KKH-5/TRP/2014<br>049/SAT-DN/BKSDA-06/IX/2013<br>037/SAT-DN/BKSDA-06/VI/2014

Bernhard Klarner, Stefan Scheu, Mark Maraun, Valentyna Krashevska	Research permits:<br>332/SIP/FRP/SM/IX/2012,<br>389/SIP/FRP/SM/X/2013,<br>19/SIP/FRP/SM/I/2015<br>145/SIP/FRP/SM/V/2013<br>16/EXT/SIP/FRP/SM/III/2014<br>Export permits:<br>S.07/KKH-2/2013<br>S.246/KKH-2/201<br>125/KKH-5/TRP/2014<br>126/KKH-5/TRP/2014
Holger Kreft, Katja Rembold, Sri Sudarmiyati Tjitrosoedirdjo	Research permits:<br>207/SIP/FRP/SM/VI/2012<br>25/EXT/SIP/FRP/SM/III/2013<br>17/EXT/SIP/FRP/SM/III/2014<br>Collection permit:<br>2266/IT/PL/2013<br>2696/IPH.1/KS.2/XI/2012
Stefanie Steinebach	Research permit:<br>264/SIP/FRP/SM/VII/2012
Ana Meijide, Alexander Knohl	Research permits<br>389/FRP/SM/II/2013<br>05/EXP/SIP/FRP/SM/II/2014
Martyna Kotowska, Dietrich Hertel, Christoph Leuschner	SIP: 206/SIP/FRP/SM/VI/2012<br>46/EXT/SIP/FRP/SM/2013<br>SPP: 2335/FRP/SM/VI/2012<br>5/TKPIPA/FRP/SM/V/2013<br>Collection permit:<br>2704/IPH.1/KS.02/X1/2012<br>Export permit:<br>49/KKH-5/TRP/2014
Anna Mareike Holtkamp	Research permit:<br>375/SIP/FRP/SM/IX/2012
Vijesh Krishna	Research permit:<br>6/TKPIPA/FRP/SM/VI/2012<br>172/SIP/FRP/SM/VI/2015

Supplementary Table 5. Sampling effort and number of taxa detected.

Variable	Method	Sub-plot replicate	Total number of taxa observed				
			Across systems	Forest	Jungle rubber	Rubber plantation	Oil palm plantation
**Trees (DBH ≥10 cm)**	All trees within the entire plot with a DBH ≥10 cm were identified and measured (position within the plot, height, DBH, crown structure)	Entire plot	482	384	199	6	3
**Understorey vegetation**	All vascular plants within the sub-plots were counted, identified and measured (height)	Five 5 x 5 m sub-plots	1505	1024	625	254	246
**Birds**	Point counts and sound recordings	No	65 (genera)	40	28	31	11
**Litter invertebrates**	Litter sieving	Three 5 x 5 m subplots	909	422	399	332	266
**Termites**	Transect surveys with searching of soil surface, leaf-litter, and tree (Note: 16 were sampled, 4 for each land-use type, in Bukit Dua Bela landscape)	No, 1 50x10m transect	30	13	21	12	9
**Ants**	Baiting and direct sampling	Five 5 x 5 m subplots	129	83	73	76	73
**Testate amoebae (protists)**	For each of the 32 plots, three cores (5 cm diameter) from the litter/fermentation layer were taken and pooled in order to account for small scale spatial variation. Testate amoebae were extracted by washing and filtering of litter material. From the final filtrate microscopic slides were prepared and testate amoebae were identified to morphospecies.	Three cores per plot	150	118	97	34	67

**Prokaryotic soil community**	16S rRNA gene analysis of bacterial and archaeal community composition (see Schneider et al. 2015)	Three sub-plots	Bacteria: 1160.411 +/- 245.566 Archaea: 113.645 +/- 39.428	Bacteria: 957.096 +/-180.031 Archaea: 121.230 +/- 38.508	Bacteria: 1052.075 +/-139.207 Archaea: 112.946 +/- 46.509	Bacteria: 1258.708 +/- 168.094 Archaea: 105.842 +/- 46.434	Bacteria: 1373.767 +/- 230.017 Archaea: 114.562 +/- 26.260
**Genetic diversity of plants**	Dominant tree species were determined using the Bitterlich-Method.	No	112	36	34	31	24
**Nutrient leaching fluxes**	Suction cup lysimeters installed into the soil at 1.5 m depth and sampled biweekly to monthly from Feb. - Dec. 2013; leaching fluxes were calculated from the monthly elements concentration and water drainage flux, estimated using the soil water module of the Expert-N model and parameterized with the measured soil, vegetation and climatic characteristics.	Two sub-plots except for oil palm plots (one sub-plot)	-	-	-	-	-
**Soil processes and functioning**	Litterbags (20 x 20 cm with 4 mm mesh size), containing 10 g dry leaf litter mixture of three tree species from one of the forest plots, were incubated in situ with one litterbag in each of the 32 plots from October 2013 to March 2014. Mass loss was calculated as the difference between the initial litter dry mass and litter dry mass remaining after 6 months and expressed as percentage of the initial leaf litter mass. Additionally, soil samples down to a depth of 10 cm were taken with a corer (5 cm diameter) at 3 subplots in each of the 32 plots. Three cores were taken from each subplot plot in order to account for small scale spatial variation. Soil samples were pooled from each set of three cores. From these soil samples, basal respiration and microbial biomass were determined by measuring O_2 consumption using an automated respirometer system. Microbial specific respiration was calculated as µl O_2 mg^{-1} C_{mic} h^{-1}.	No, for litterbags.<br><br>Three sub-plots for basal respiration, microbial biomass, microbial specific respiration.	-	-	-	-	-

**Soil sampling and fertility characteristics**	Soil samples were taken in the top 0.1 m depth, except for net N mineralization which was in the top 0.05 m. Exchangeable bases and Al were determined from air-dried, 2-mm sieved soils, using the standard 1M KCl percolation method, and extractable P using the Bray 2 extraction method; extracts were analyzed using an ICP-AES. Soil organic C was measured down to 2-m depth; this was determined from air-dried, sieved and ground soils using a CN analyzer. ^{15}N natural abundance was analyzed from air-dried, sieved and finely ground soils using isotope ratio mass spectrometry. Net N mineralization was measured using the buried bag method on freshly sampled intact soil cores which were incubated in situ for 7 days; soils were then extracted in situ with 0.5 M K_2SO_4. Extracts were immediately frozen for transport and analyzed using continuous flow injection colorimetry.	Ten sub-plots, except for net N mineralization with two sub-plots	-	-	-	-	-
**Stability in climatic conditions**	Weather stations in the center of the plots measuring hourly air temperature and humidity and soil temperature and moisture	N0	-	-	-	-	-
**Above- and belowground biomass and carbon stock**	Stand structural data of all trees, palms and lianas with DBH > 10 cm and biomass calculation using allometric equations; plus understory trees with a DBH of 2 - 9.9 cm<br>Fine root biomass down to 50 cm	Small trees < 9.9 cm DBH on two subplots	-	-	-	-	-
**Net primary productivity**	Litter fall measurements (16 litter traps per plot, leaf count in oil palm)<br>Woody biomass production (stem increment measurements in 40 trees per plot, oil palm height increment)<br>Fine root production measured with ingrowth cores (n=16)	16	-	-	-	-	-

Supplementary Notes

Supplementary Note 1: Historical and institutional background

Today's land-use patterns in Jambi province result from a complex interplay of concurrent and often ambivalent, constantly renegotiated, institutional frameworks, which have to be analyzed against their historical background. The first set of rules, which changed the local system of property rights and access to land, evolved with the Dutch colonial administration. Initially interested in controlling the region's spice trade, the Dutch soon implemented a cultivation system under which certain cash crops like coffee, sugar, tea, cinnamon, tobacco, as well as silk were produced for the world market (2). In 1865, the Dutch colonial forestry law was applied in the East Indies. Together with the Dutch Agrarian Law adopted five years later, this set the legal framework for large-scale resource exploitation, agricultural intensification and plantation economy.

When the Dutch took over colonial rule in Jambi in 1904, land use was dominated by small scale agroforest systems, where different kinds of root crops and upland rice were grown in swidden cultivation. This extensive shifting cultivation system produced mosaic landscapes, where forest remnants and agricultural lands were integrated with each other. These local land use and land tenure systems were defined as "beschikkingsrecht" in Dutch – or 'right of use' as the closest translation. Areas not under cultivation (including fallows with secondary forests and shrublands) were declared as "woeste grond" (Dutch for 'uncultivated land') and as such were a property of the state (3). As the "beschikkingsrecht" did not have the character of ownership, it easily came under control of the state or the Dutch government. These policies abolished the former concepts of local land use and the complex concepts of different kinds of forests in relation to their usage by the population. Instead, it created "no mans" land that conceptually divide the society from their natural environment.

With the support of foreign capital, uncultivated land could either be assigned as leasehold for 75 years or as concession to private corporations for 99 years (4). With the expansion of the Dutch

colonial power on the Indonesian archipelago, plantation agriculture was widely introduced in Sumatra. Even more important than the rising production of local crops was the introduction of rubber in Jambi in 1904 (5). Rubber cultivation boomed from 1910 onwards with its heyday in the 1920s, due to an increased international demand and high rubber prices (6, 7). At that time, almost the entire land area of Jambi province was planted with rubber trees. The planting of rubber trees boomed and even with a local population density, according to Nasruddin (5) in 1935 around 44,000 people owned as much as 67 million rubber trees. Rubber was adopted widely in smallholdings by the local population in the studied landscapes (sedentary Batin Sembilan and Jambi Melayu). Rattan as traditional forest product was cultivated as well and was put aside when rubber business went well, but used as income when rubber prices declined during the economic depression in 1935/1936. Rattan was planted along the rivers as it was easy to harvest later using a boat. It could be harvested at the age of 6 years, as for rubber. The introduction of rubber determined Jambi's economy and environmental policies until today. The Dutch also successfully drilled for oil at the Harapan landscape.

Oil palm plantations were developed mainly in north Sumatra from 1911 onwards (8) and usually operated by private Dutch companies, which held the needed financial and workforce inputs (8). European laws and large-scale crop production by foreign corporations did not only induce major changes in land use but also restricted local populations access to the land, and thus conceptually turned the landscapes from socio-cultural systems into mere agricultural production systems.

After the Indonesian independence, the Basic Agrarian Law (BAL) from 1960 and the Forestry Law from 1967 set the legal framework for further deforestation and agricultural intensification. Together with the promotion of logging and the completion of the trans-Sumatra highway, land-use schemes in Jambi province changed massively, and in the 1970s almost the entire forested area of Jambi province was allocated as concession areas at the cost of local extensively-managed agroforest systems (9, 10).

The years following the collapse of the Soeharto regime in 1998 were characterized by a far reaching and rapid decentralization process. With the regional autonomy law (1999), administrative and regulatory authority was transferred from the national government to provincial, district and municipal governments, followed by a series of forestry sector reforms allowing district governments and local communities a greater role in forest management (11). “Within this context, district officials suddenly found that it was politically feasible to assert far-reaching administrative authority over forest resources located within their jurisdictions, and many moved aggressively to do so” (11). New district development strategies, based mainly on the exploitation of forest resources, were formulated, overlapping often with national parks or conservation areas and with concession boundaries issued by the national government. “As a result, many local regulations conflict with higher-level policies and laws, while increased decision-making powers and the quest for locally generated revenues have led to indiscriminate licensing for inappropriate forest conversion” (12). The rights of indigenous people and local communities were limited or extinguished as the Indonesian government interprets the Indonesian Constitution as granting the right to manage all natural resources to the benefit of the nation (8, 13). Overall, these state policies heavily impacted local people’s livelihood systems and pushed smallholders’ land-use systems from agroforestry-based subsistence to monoculture cash-crop production.

Supplementary References

1. Brown C (2011) A short history of Indonesia. The unlikely nation? Talisman Publishing Pte Ltd, Singapore.
2. Haughton J, Khandker SR (2009) *Handbook on poverty and inequality*. The International Bank for Reconstruction and Development/The World Bank, Washington DC. p. 104-105
3. von Benda-Beckmann F, von Benda-Beckmann K (2010) Unity and Diversity. Multiple citizenship in Indonesia. *Cultural Diversity and the Law. State Responses from Around the World,* eds. Foblets MC, Gaudreault-Desbiens JF, DundesRenteln A (Brussels), p. 889-917.
4. Löffler U (1996) Land Tenure Developments in Indonesia. Study for the guiding principles. The importance of Land Tenure in Development Cooperation. GTZ.
5. Nasruddin AM (1989) *Jambi dalam sejarah nusantara 692 – 1949 M*. (Perpustakaan Musium Nasional Jambi).
6. Feintrenie L, Levang P (2009) Sumatra’s rubber agroforests: Advent, rise and fall of a sustainable cropping system. *Small-scale Forestry* 8(3):323–335.
7. Locher-Scholten E (2003) *Sumatran sultanate and colonial state. Jambi and the rise of Dutch imperialism, 1830–1907*. (Ithaca, NY: Southeast Asia Program Publications, Cornell University).

8. Jiwan N (2013) The political ecology of the Indonesian palm oil industry. *The palm oil controversy in Southeast Asia. A transnational perspective*, eds. Pye O, Bhattacharya J. (Singapore: Institute of Southeast Asian Studies). pp. 48–75.
9. Martini E, et al. (2010). Rubber agroforests and governance at the interface between conservation and livelihoods in Bungo district, Jambi province, Indonesia. World Agroforestry Centre, Working paper 124, Bogor, Indonesia.
10. Colchester M, Anderson P, Firdaus AY, Hasibuan F, Chao S (2011) *Human rights abuses and land conflicts in the PT Asiatic Persada concession in Jambi. Report of an independent investigation into land disputes and forced evictions in a palm oil estate*. Bogor, Indonesia: Forest Peoples Programme, SawitWatch, HuMa.
11. Barr C, et al. (2006) *Decentralization of forest administration in Indonesia: Implications for forest Sustainability, economic development and community livelihoods*. Bogor, Indonesia: Center for International Forestry Research.
12. Indrarto GB, et al. (2012) *The context of REDD+ in Indonesia: drivers, agents, and institutions*. Center for International Forestry Research, Bogor, Indonesia.
13. Anderson, P (2013) Free, prior and informed consent? Indigenous peoples and the palm oil boom in Indonesia. *The palm oil controversy in Southeast Asia. A transnational perspective*, eds. Pye O, Bhattacharya J. (Singapore: Institute of Southeast Asian Studies) pp. 244–258.
